# Beyond labeling: differential Ac_4_ManNAz dosing as a tool for functional manipulation of mesenchymal stem cells

**DOI:** 10.1093/rb/rbag044

**Published:** 2026-03-09

**Authors:** Xueying Zhao, Suqing Li, Xingyu Jiang, Yuyang Ma, Kewei Fan, Luzhong Zhang, Xin Liu, Yumin Yang

**Affiliations:** Medical School of Nantong University, Key Laboratory of Neuroregeneration of Jiangsu and Ministry of Education, Co-innovation Center of Neuroregeneration, Nantong University, 19 Qixiu Road, Chongchuan District, Nantong, Jiangsu Province 226001, China; Medical School of Nantong University, Key Laboratory of Neuroregeneration of Jiangsu and Ministry of Education, Co-innovation Center of Neuroregeneration, Nantong University, 19 Qixiu Road, Chongchuan District, Nantong, Jiangsu Province 226001, China; Medical School of Nantong University, Key Laboratory of Neuroregeneration of Jiangsu and Ministry of Education, Co-innovation Center of Neuroregeneration, Nantong University, 19 Qixiu Road, Chongchuan District, Nantong, Jiangsu Province 226001, China; Medical School of Nantong University, Key Laboratory of Neuroregeneration of Jiangsu and Ministry of Education, Co-innovation Center of Neuroregeneration, Nantong University, 19 Qixiu Road, Chongchuan District, Nantong, Jiangsu Province 226001, China; Medical School of Nantong University, Key Laboratory of Neuroregeneration of Jiangsu and Ministry of Education, Co-innovation Center of Neuroregeneration, Nantong University, 19 Qixiu Road, Chongchuan District, Nantong, Jiangsu Province 226001, China; Medical School of Nantong University, Key Laboratory of Neuroregeneration of Jiangsu and Ministry of Education, Co-innovation Center of Neuroregeneration, Nantong University, 19 Qixiu Road, Chongchuan District, Nantong, Jiangsu Province 226001, China; The Third Clinical Medical College, Affiliated Hospital of Integrated Traditional Chinese and Western Medicine, Nanjing University of Chinese Medicine, No. 100 Cross Street, Hongshan Road, Qixia District, Nanjing, Jiangsu Province 210028, China; Medical School of Nantong University, Key Laboratory of Neuroregeneration of Jiangsu and Ministry of Education, Co-innovation Center of Neuroregeneration, Nantong University, 19 Qixiu Road, Chongchuan District, Nantong, Jiangsu Province 226001, China

**Keywords:** Ac_4_ManNAz, metabolic labeling, mesenchymal stem cell, immunoregulatory, physiology

## Abstract

Bioorthogonal chemistry technology serves as a powerful tool for mesenchymal stem cell (MSC) transplantation by utilizing metabolic engineering for direct cell modification either *in vivo* or *in vitro*. However, the unclear effects of varying concentrations of non-natural metabolic monosaccharides on labeling efficiency, functional impact and safety pose significant challenges for clinical translation. Herein, we screened the concentration-dependent effects of the metabolic labeling agent Ac_4_ManNAz (10–100 µM) on the functionality of MSC. Visualizing azido groups on the cell surface through bioorthogonal reactions confirmed that labeling efficiency positively correlated with the concentration of Ac_4_ManNAz. Interestingly, treatment of MSC with 50-µM Ac_4_ManNAz enhances their immune regulatory function. Mechanistically, 50-µM Ac_4_ManNAz remodels the extracellular matrix of MSC by downregulating decorin (DCN) expression to alleviate TGF-**β** inhibition, simultaneously upregulating serglycin (SRGN) expression to activate and stabilize TGF-**β**. This dual action results in increased secretion of TGF-**β**, thereby augmenting the immune regulatory capacity of MSC. Nevertheless, at the level of cellular motility, treatment with 50-µM Ac_4_ManNAz significantly impairs the migratory capacity of MSC. Overall, this study clearly defined the concentration effect of non-natural monosaccharides on MSC biological functionality for clinical applications of this technology.

## Graphical Abstract

**Graphical Abstract:**
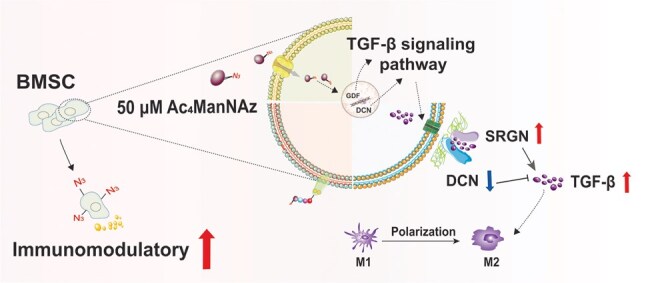

## Introduction

Mesenchymal stem/stroma cells (MSCs) have demonstrated significant therapeutic potential in clinical trials for cell-based therapies, with applications spanning a diverse spectrum of diseases including autoimmune disorders, neurological conditions, cardiovascular diseases and oncology [1–3]. Clinical trials have demonstrated the safety of high-dose MSC administration and reported favorable outcomes in certain cases [4, 5]. However, the therapeutic potential of MSC-based therapies in addressing all disease-associated challenges is constrained by limitations in delivery efficiency and inherent functional properties [6]. To overcome these limitations, various strategies have been applied to enhance delivery precision and augment the performance of MSC. Current techniques include biomaterial-based encapsulation, surface engineering and genetic modification to optimize therapeutic efficacy [7, 8]. For example, biomaterial encapsulation has been employed to prolong MSC survival and improve retention [9]. Concurrently, cell surface engineering demonstrates multiple advantages, including stem cell tracing [10], functional induction [11] and targeted delivery of stem cells to specific tissues mediated by bioorthogonal reactions [12, 13]. In these methodologies, bioorthogonal technology showcases distinct advantages, including non-invasive labeling, superior biocompatibility, remarkable specificity and the capacity for prolonged fate monitoring [14]. However, the current choice of metabolic monosaccharide concentration for cell labeling shows considerable variability. It is imperative to elucidate the impact of azido modification concentration on stem cell functionality, as this understanding bears significant implications for a myriad of applications.

Tetra-acetylated N-azidoacetyl-D-mannosamine (Ac_4_ManNAz), a monosaccharide analog, is widely used for cell labeling, glycoproteomic analysis and tissue engineering field [15, 16]. Studies have demonstrated that the labeling efficiency of azido-sugars is significantly higher in adipose tissue-derived human mesenchymal stem cells when compared to tetra-acetylated N-azidoacetyl-galactosamine (Ac_4_GalNAz) or tetra-acetylated N-azidoacetyl-glucosamine (Ac_4_GlcNAz) [17]. The concentration of Ac_4_ManNAz is frequently utilized in a cross-concentration gradient ranging from 0 to 100 µM for optimization experiments [18]. However, the application of Ac_4_ManNAz-based metabolic labeling in MSC for therapy often prioritizes labeling efficiency, overlooking the potential effects of azido-sugar concentration on MSC physiological functions [19]. Actually, incorporating the azido groups into cellular glycoproteins via Ac_4_ManNAz occurs mainly through post-translational modification. Once integrated into cellular glycans, the azido-sugar is converted to azido-sialic acid derivatives and subsequently utilized in N-linked glycosylation of cell surface proteins [20]. This process involves complex site-specific heterogeneity that regulates diverse cellular physiological properties [21]. Although azido groups offer significant advantages for bioconjugation due to their minimal perturbation of native protein structures and low reactivity with endogenous biomolecules, emerging evidence indicates that the concentration of Ac_4_ManNAz used in metabolic labeling can influence critical physiological characteristics of the treated cells [22, 23]. In light of these considerations, we ultimately selected Ac_4_ManNAz as the azido-labeled monosaccharide and implemented a large-span concentration gradient for our screening system involving MSCs.

The therapeutic efficacy of MSC can be attributed to four fundamental mechanisms, including homing capacity, multilineage differentiation potential, paracrine activity and immunomodulation [24]. Evidence suggests that the role of MSCs differentiating into specific functional cells at injury sites for direct tissue repair is minimal. Instead, their efficacy primarily stems from robust paracrine and immune regulatory functions [25, 26]. MSCs secrete various nutritional factors, chemokines and immune regulators [e.g. insulin growth factor (IGF), interleukin-6 (IL-6), transforming growth factor-beta (TGF-β)] that reshape the local microenvironment at injury sites. This process inhibits excessive inflammation and promotes endogenous cell proliferation and tissue repair, thereby achieving therapeutic goals indirectly [27]. Consequently, their paracrine activities reveal remarkable clinical promise [28–31]. The inflammatory milieu [32], hypoxic conditions [33], the intrinsic properties of delivery materials [34, 35] and physicochemical factors such as pH levels and hyperglycemic environments [36] can all significantly influence the secretory functions of MSC. Hence, it is imperative to explore the influence of metabolic labeling reagent concentration on the viability and functional capacities of MSCs for subsequent clinical translation.

Transforming growth factor-beta (TGF-β) is a key immune regulatory cytokine secreted by MSC [37]. TGF-β has three gene products: TGF-β1, TGF-β2 and TGF-β3, all expressed in an inactive form known as latent TGF-β (L-TGF-β). This consists of mature TGF and latency-associated peptide (LAP). After secretion, the TGF-β complex can either bind within the extracellular matrix (ECM) or attach to the cell surface and participate in the activation process of cells [38, 39]. The activation process is crucial for cellular function and requires specific stimuli, such as integrin-mediated mechanical forces, proteolytic hydrolysis or acidic environments (to disrupt molecular bindings). This release enables active TGF-β to perform key immunoregulatory functions such as modulating macrophage polarization [40]. Reports show that increasing Ac_4_ManNAz concentrations to 50 µM significantly alters gene expression related to cytokine interactions, chemokines and ECM receptor interactions in human umbilical cord blood-derived endothelial progenitor cells (hUCB-EPC) [22]. Thus, it is reasonable to hypothesize that Ac_4_ManNAz concentration may influence the secretion and activation of TGF-β factors by MSC, opening new paths for effective MSC therapies.

In this study, we evaluated the effects of various Ac_4_ManNAz concentrations on the physiological properties and biological functions of bone marrow-derived MSCs. We systematically analyzed labeling efficiency, cell viability, migration ability and immunoregulatory properties of MSCs after treatment with Ac_4_ManNAz. The metabolic labeling efficiency of azido groups on the MSC surfaces was found to be concentration-dependent. Treatment with 100 µM of Ac_4_ManNAz decreased MSC viability, as shown by CCK-8 and lactate dehydrogenase (LDH) release assays. MSCs treated with 50-µM Ac_4_ManNAz exhibited reduced migratory capacity, diminished mitochondrial membrane potential and caused morphological changes. Interestingly, it enhanced the ability of MSCs to promote M1 to M2 polarization. Further investigation revealed that treatment with 50-µM Ac_4_ManNAz altered ECM components of MSCs in two main ways: first, downregulation of decorin (DCN) alleviated its inhibitory effect on TGF-β proteins; second, homeostatic expression of serglycin (SRGN) facilitated TGF-β protein activation and stabilization. These combined effects led to increased active TGF-β secretion and enhanced immunoregulatory capabilities of MSCs. Our findings suggest that broad screening of Ac_4_ManNAz on MSCs can guide more detailed investigations and expand the applications of metabolic labeling-based MSC therapy in immune-related diseases.

## Materials and methods

### Cell isolation and culture

Primary bone marrow-derived MSCs (BMSCs) were obtained from 3-week-old female Sprague–Dawley (SD) rats (Laboratory Animal Center of Nantong University; Approval ID: S20250516-002) and rats weighing 50–70 g were used in the following experiments. The MSCs were cultured according to established protocols with minor adjustments [41]. Briefly, the rats were executed under anesthesia. Then, sterilized with 75% ethanol and peeled off the femur and tibia from the hind legs. Cut off both ends of the bones and rinse the marrow cavity repeatedly with a 1-mL injection syringe. After being filtered with a 200-mesh strainer, the cell suspension was centrifuged at 160–200 g for 5 min. Then the single-cell suspension of BMSCs was prepared by resuspending the precipitate with the low-glucose Dulbecco’s modified Eagle’s medium (Hyclone, USA). Cells were cultured in a T25 culture flask (NEST biotechnology Co., Ltd., China) in the proliferation medium containing 15% fetal bovine serum (Gibco, USA), 1% penicillin-streptomycin (NCM Biotech, China) and were cultured at 37°C under an atmosphere of 5% CO_2_. The cells were passaged when reaching the confluence at 80%, and the passage ratio was 1:2 every 7 days. MSCs at passage 3–5 were used in the following experiments.

### Azido-tagging cells and characterization

The MSCs were seeded in a 24-well plate at a density of 5 × 10^4^ cells/well and cultured at 37°C under an atmosphere of 5% CO_2_ for 4 h. Then, the cells were treated with different concentrations of Ac_4_ManNAz (final concentrations were 0, 10, 20, 50 and 100 μM, respectively) in cell culture medium for 48 h to ensure the N_3_ groups were fully tagged to the cells surface by glycan metabolism. The expression of N_3_-groups on MSCs’ surface was detected by DBCO-Cy3 (50 μM) fluorescent staining through strain-promoted alkyne-azide cycloadditions click reactions. Briefly, after Ac_4_ManNAz treatment, MSCs were washed with PBS and incubated with DBCO-Cy3 in the dark for 30 min at 37°C. Then, the MSCs were fixed by 4% paraformaldehyde (PFA, Beyotime, China) solution for 30 min at room temperature (RT) or 4°C overnight. Further, the PFA was moved and washed with PBS for three times, then the samples were blocked with 5% bovine serum albumin (Sangon Biotech, China) containing 0.3% Triton X-100 for 1 h at RT. Subsequently, the cell nuclei were counterstained with DAPI reagent for 5–10 min. Finally, the images were collected using a fluorescence microscope (Axioscope 5, ZEISS, Germany) and quantitatively analyzed using ImageJ software.

### Cell viability analysis

To evaluate the survival rate of MSCs after being treated with azido-sugar. MSCs with a density of 1 × 10^4^ cells/well were seeded on 96-well cell culture plates and incubated in an incubator for 4 h. Subsequently, azido-sugar with different concentrations (0, 10, 20, 50 and 100 μM, respectively) was added to the culture medium and continuously cultured for 24 or 72 h. The CCK-8 experiment was performed by adding the CCK-8 kit into the culture medium at a volume ratio of 10% and incubating at 37°C for 4 h. The absorbance of the medium at 450 nm was tested by a microplate reader (BioTek, USA). Cell death rate was evaluated by LDH cytotoxicity assay kit (Beyotime, China) according to the manufacturer’s protocol at 24 h.

### Cell migration and invasion abilities

The scratch assay was performed to evaluate the MSCs’ migration ability following the Ac_4_ManNAz treatment. Briefly, when MSCs/N_3_-MSCs reached 80–90% confluency, a straight scratch on the monolayer cell was created using a 200-μL pipette tip and gently washed with PBS. Then, serum-free medium was added to each well. Cell migration was assessed by capturing optical photographs of the scratch edge 24 h post-scratch using an inverted microscope (Leica, Germany). Quantitative analysis was performed on the captured images using ImageJ software.

For the trans-well assay, 5 × 10^4^ MSCs or MSCs treated with 10 or 50 μM Ac_4_ManNAz (N_3_-MSCs) were pipetted to the upper chamber of each well and supplied with serum-free medium. The bottom chamber was supplied with complete medium. After 24 h of culture, a transmembrane filter was performed, and a crystal violet staining assay was used to calculate the cell number.

### Mitochondrial morphology analysis

Morphology of mitochondria in MSCs before and after azido-sugar treatment was observed using a transmission electron microscope (TEM; Hitachi, Japan). In general, cells were treated as *above-described protocol*, while the cell density was 5 × 10^5^ cells/well. MSCs/N_3_-MSCs were harvested 2 days after treatment, and fixed with 2.5% glutaraldehyde in agarose at 4°C overnight for subsequent observation.

### Mitochondrial membrane potential analysis

For mitochondria membrane potential (MMP) test, JC-1 (5,5′,6,6′-tetrachloro-1,1′,3,3′-tetraethyl-imidacarbocyanine iodide; Beyotime, China) staining and TMRE (tetramethylrhodamine, ethyl ester; Beyotime, China) assay was performed. Briefly, MSCs were grown in glass slides in 24-well plates and treated with 10 or 50 μM Ac_4_ManNAz for 48 h. After washing with PBS for three times, MSCs/N_3_-MSCs were incubated with 10 μg/mL JC-1 dye for 20 min at 37°C according to the manufacturer’s instructions. For the TMRE assay, MSCs/N_3_-MSCs were incubated with TMRE Probe and Hoechst 33342 (CellorLab, China) for 15 min at 37°C. Finally, fluorescence images were taken by fluorescence microscopy (ZEISS, Germany). The ratio of red fluorescence JC-1 aggregates and green JC-1 monomers was calculated using ImageJ software.

### Mitochondrial energy metabolism analysis

BMSCs were seeded into Seahorse XF96 microplates at 8 × 10^3^ cells/well with (10 and 50 μM) or without Ac_4_ManNAz treated for 48 h. Before testing, cells were washed with XF Assay medium (Agilent, USA) for two times and subsequently incubated at 37°C without CO_2_ for 1 h. Oxygen consumption rates (OCRs) were measured by a Seahorse XFe24 Analyzer (Seahorse Bioscience) following sequential treatment with oligomycin (2.5 μM), Carbonyl cyanide-4-(trifluoromethoxy)phenylhydrazone (FCCP, 2 μM), and rotenone plus antimycin A (R/A, 0.5 μM). Basal respiration was calculated as the OCR before the first injection minus the lowest OCR after the final injection. The maximal respiratory capacity was defined as the peak OCR observed following FCCP administration, minus the basal respiration. ATP production was calculated by subtracting post-oligomycin OCR from basal OCR. Finally, spare respiratory capacity was determined by taking the difference between maximal respiration and basal respiration.

### Enzyme-linked immunosorbent assay analyses

An enzyme-linked immunosorbent assay (ELISA) was performed to assess cytokine expression differences between the N_3_-MSCs group and the MSCs group using an ELISA kit (TGF-β, Elabscience, China) (IGF-2, MEIKE, China). Briefly, 48 h after azido-sugar treatment, MSCs/N_3_-MSCs were treated with serum-free medium and continued to be cultured for 48 h. Subsequently, the culture supernatant from MSCs/N_3_-MSCs was collected as MSC-conditioned medium (CM)/N_3_-MSCs-CM. Then, the standard working solution and samples were added to the ELISA plates pre-coated with TGF-β antibody. After incubating at 37°C for 90 min, the biotinylated detection Ab working solution was added and further incubated for 60 min at 37°C, followed by a washing protocol to remove excess reagent. Subsequently, the horseradish peroxidase (HRP) conjugate working solution was added to each well for 30 min at 37°C. The wash process was repeated five times, and substrate reagent was added to each well for 15 min at 37°C. Finally, the reaction in each well was stopped by the stop solution. The optical density was determined immediately using a microplate reader at 450 nm.

### Analysis of the immunomodulatory capacity of macrophages

RAW264.7 cells were seeded on six-well plates (1 × 10^5^) and incubated for 4 h. Cells were then treated with MSCs-CM/N_3_-MSCs-CM (adding 100-ng/mL lipopolysaccharide (LPS) and 10-ng/mL IFN-γ), which was added to each well for 24 h at 37°C. Immunofluorescent (IF) staining for M1 marker CD86 and M2 marker CD206. Quantitative polymerase chain reaction (qPCR) assay was performed to analyze the expression of pro-inflammatory and anti-inflammatory cytokines.

In a cell-to-cell co-culture system, macrophages and MSCs/N_3_-MSCs were plated in six-well plates at a density of 2 × 10^5^ cells per well to achieve co-culture (macrophages: MSC/N_3_-MSC = 3:1, adding 100-ng/mL LPS and 10 ng/mL IFN-γ). After 24 h, cells were harvested for further analysis.

### Real-time qPCR

Total RNAs were extracted from cells using Trizol reagent following the manufacturer’s instructions. After measuring the concentration of mRNAs, cDNAs were synthesized using a reverse transcription reaction with a reverse kit (Takara, Japan). SYBR Green-based (Roche, Swit) qPCR was performed on an Applied Biosystems PRISM 800 Fast Real-time PCR system to evaluate mRNA expression levels. The primers were synthesized by Sangon Biotech, China. The primer sequences are listed in Supplementary Table S1.

### Transcriptomic analysis of MSCs

The MSCs/N_3_-MSCs were harvested for the transcriptomic studies after 48 h of treatment with/without azido-sugars (10 and 50 μM). Briefly, total RNAs were extracted with Trizol reagent (Sigma, USA) and stored at −80°C until use. After being qualified, 1-μg total RNA in each group was used for subsequent library preparation. The resulting mRNA was purified with Oligo (dT) beads and fragmented by divalent cations and high temperature. Random primers to synthesize first- and second-strand cDNAs. Then, the purified double-stranded cDNA was repaired at both ends and had adaptors added to both ends through T-A ligation reaction. DNA clean beads were used to screen the adaptor-ligated DNA based on size. Samples were amplified by PCR, and the PCR product was purified. Finally, the constructed libraries with different indices were sequenced using an Illumina HiSeq with 2 × 150 paired-end sequencing strategy.

### Western blotting assay

The whole proteins of cells and tissue extracts were prepared by lysing agent containing proteinase inhibitors (Beyotime, China). After that, extracts were centrifuged at 14 000 g for 15 min at 4°C. The protein concentration in the supernatant was quantified using a BCA kit (Beyotime, China). After denaturation, total proteins were separated by 10% SDS-polyacrylamide gel electrophoresis, and then transferred to polyvinylidene difluoride membranes by a Semi-Dry instrument (Bio-rad, Inc, USA). Next, the nonspecific sites were blocked with 5% defatted milk (Sangon Biotech, China) at RT for 1.5 h, and the primary antibody solution was incubated with the membranes at 4°C for at least 12 h. Then, remove the primary antibody, wash the membranes in Tris-buffered saline with Tween 20 (TBST) three times, and then incubate the membranes with the corresponding horseradish peroxidase-conjugated secondary antibody according to the species of primary antibody. Finally, after washing with TBST three times, immunopositivity bands were visualized using an electrochemiluminescence (ECL) western blotting (WB) detection system (Amersham ECL Plus, GE Healthcare).

Antibodies we used here are as listed: SRGN (1:1000; Santa Cruz Biotechnology, USA), TGF-β (1:800; Santa Cruz Biotechnology, USA; 1:1000, Selleck, China), α-Tubulin (1:10 000; Proteintech, China), GAPDH (1:20 000; Proteintech, China), Goat anti-mouse IgG (H + L) (1:1500; Beyotime, China), Goat anti-rabbit recombination IgG (H + L) (1:1000; Beyotime, China).

### SRGN siRNA transfection and rescue assay

N_3_-MSCs were transfected with SRGN siRNA diluted in transfection medium according to the manufacturer’s protocol. The efficiency of SRGN knockdown was evaluated by qPCR assay. For the rescue assay, the experimental group was treated with SRGN siRNA for 24 h before adding 50 μM Ac_4_ManNAz-sugar. After 48 h, protein was harvested from each group for WB analysis. The SRGN siRNA primer sequences are listed in Supplementary Table S2.

### Cellular differentiation potential analysis

To investigate the impact of various concentrations of Ac_4_ManNAz on the differentiation capabilities of MSCs, we conducted a tri-differentiation experiment on MSCs before and after treatment with Ac_4_ManNAz. This included assessments of adipogenic, osteogenic and chondrogenic differentiation potential of MSCs/N_3_-MSCs. In general, MSCs with or without Ac_4_ManNAz treatment were incubated in adipogenic medium, osteogenic culture medium and chondrogenic medium (Servicebio, China) for 7 days, respectively. Subsequently, qPCR analysis was conducted on specific markers corresponding to the differentiation direction, primers were listed in Supplementary Table S1. Oil Red O staining (Servicebio, China) is utilized to characterize cells undergoing adipogenic differentiation. The Alizarin Red S staining (ARS; Servicebio, China) is utilized for the characterization of osteogenic differentiation in MSCs, and Alcian Blue staining (Servicebio, China) for chondrogenic differentiation at Day 18.

### Statistical analysis

Standard statistical tests were conducted using GraphPad Prism 9 (Graph-Pad Software Inc., La Jolla, CA, USA). The unpaired two-tailed Student’s *t*-test and one-way analysis of variance were used to compare the mean values of two independent samples and for multiple comparisons, respectively. The data are presented as mean ± SD. The results were considered significant at *P < 0.05, **P < 0.01, ***P < 0.001, ****P < 0.0001, *n* ≥ 3.

## Results and discussion

### Impact of Ac_4_ManNAz on the fundamental characteristics of MSCs

To evaluate the metabolic labeling efficiency and safety of BMSCs after treatment with Ac_4_ManNAz. First, BMSCs were isolated and characterized according to the previously published methodology [42]. The phenotype of MSC was confirmed by morphological observation, IF staining, and qPCR analysis. Optical images revealed that the cells maintained a fibroblast-like morphology at passages 0, 2 and 4 (p_0_, p_2_ and p_4_). IF staining demonstrated positive expression of the typical MSC surface markers CD29 and CD90, while CD34 showed negative expression (Supplementary Figure S1A). The MSCs expressed the MSC markers at the gene level, specifically CD29 and CD90, while showing no expression of CD11b and CD45, as determined by qPCR (Supplementary Figure S1B). After 2 weeks of adipogenic and osteogenic differentiation induction, MSCs exhibited characteristic differentiation phenotypes. Adipogenic differentiation was confirmed by the presence of cytoplasmic lipid droplets visualized through Oil Red O staining. Additionally, osteogenic differentiation was evidenced by detectable calcium deposition as indicated by positive ARS (Supplementary Figure S1C).

**Figure 1:**
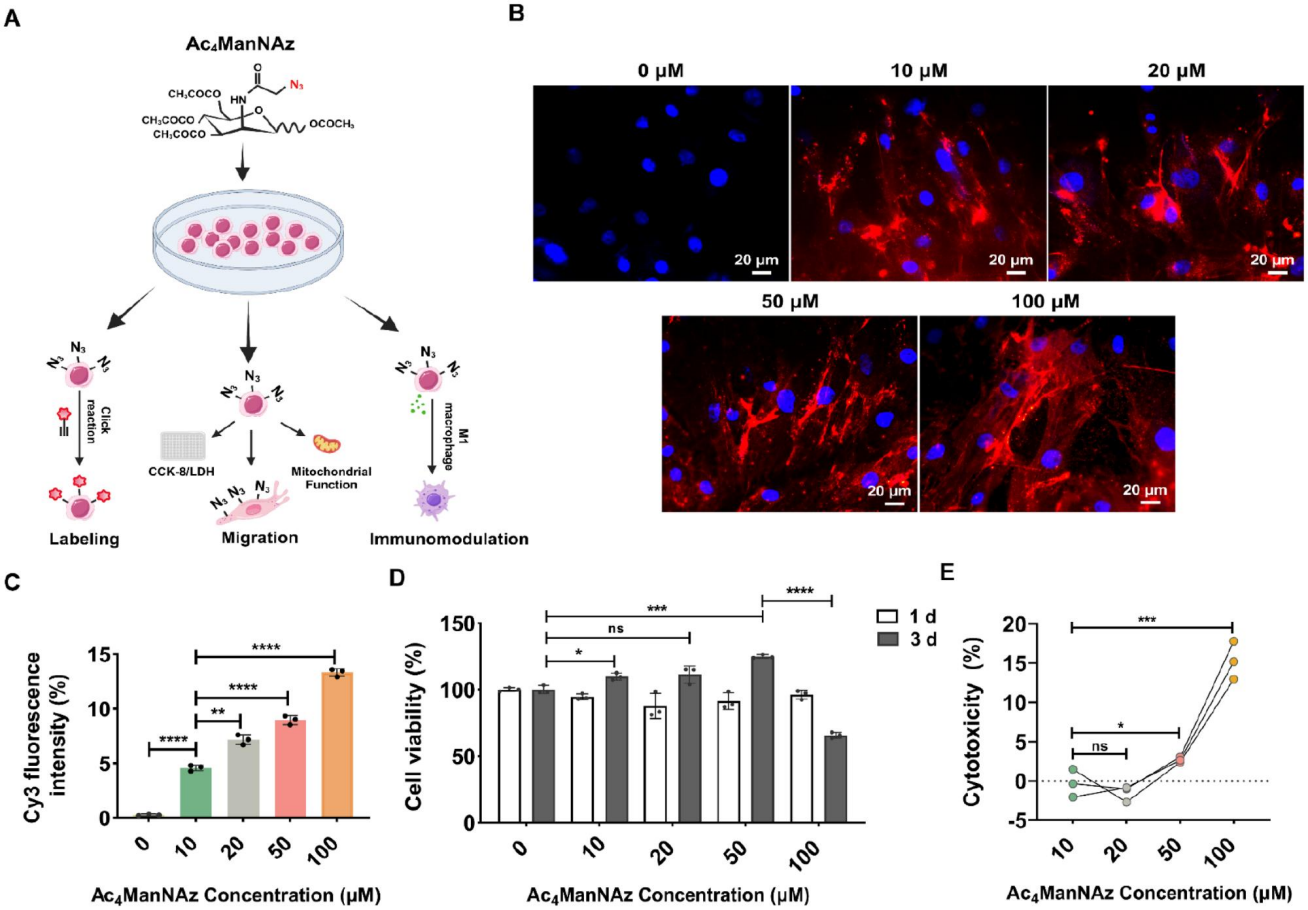
Analysis of metabolic labeling efficiency and viability of Ac_4_ManNAz-treated BMSCs. (**A**) A schematic overview of the evaluation criteria for MSCs following treatment with Ac_4_ManNAz. (**B**) Visualization of azido groups on MSCs using DBCO-Cy3. Scale bar, 20 μm. (**C**) Relative fluorescence intensity of Cy3 (*n* = 3). (**D**) Quantitative analysis of MSCs viability by CCK-8 assay following azido-sugar treatment (*n* = 3). (**E**) The cytotoxicity was analyzed by LDH assay (*n* = 3). data are expressed as mean ± SD; *P < 0.05, **P < 0.01, ***P < 0.001, ****P < 0.0001 and ns represent *P* > 0.05.

Labeling efficiency, cell viability, migration capability and immunoregulatory property of MSCs were evaluated after treatment with various concentrations of Ac_4_ManNAz (Figure 1A). First, azido groups on MSC membranes were detected via biorthogonal copper-free click chemistry reaction using Cy3-labeled dibenzyl cyclooctyne (DBCO-Cy3) (Figure 1B). Relative fluorescence intensity analysis confirmed dose-dependent incorporation of abiotic azido groups on the cell membrane (Figure 1C). Cell viability was assessed using the CCK-8 assay at 1- and 3-day intervals, revealing that a moderate azido-sugar concentration increased cellular activity by Day 3, while 100-μM treatment significantly decreased the viability of MSCs (Figure 1D). Our observation contradicts earlier reports suggesting that MSCs tolerate Ac_4_ManNAz concentrations of up to 250 μM [43, 44]. This discrepancy may be attributed to variations in the sources of MSCs and their respective culture conditions. LDH assays showed a progressively higher cell death rate in MSCs treated with Ac_4_ManNAz concentration at 100 μM (Figure 1E). Therefore, 100 μM was not adopted for subsequent experiments.

### Evaluation of adaptability of MSCs after treatment with Ac_4_ManNAz

**Figure 2:**
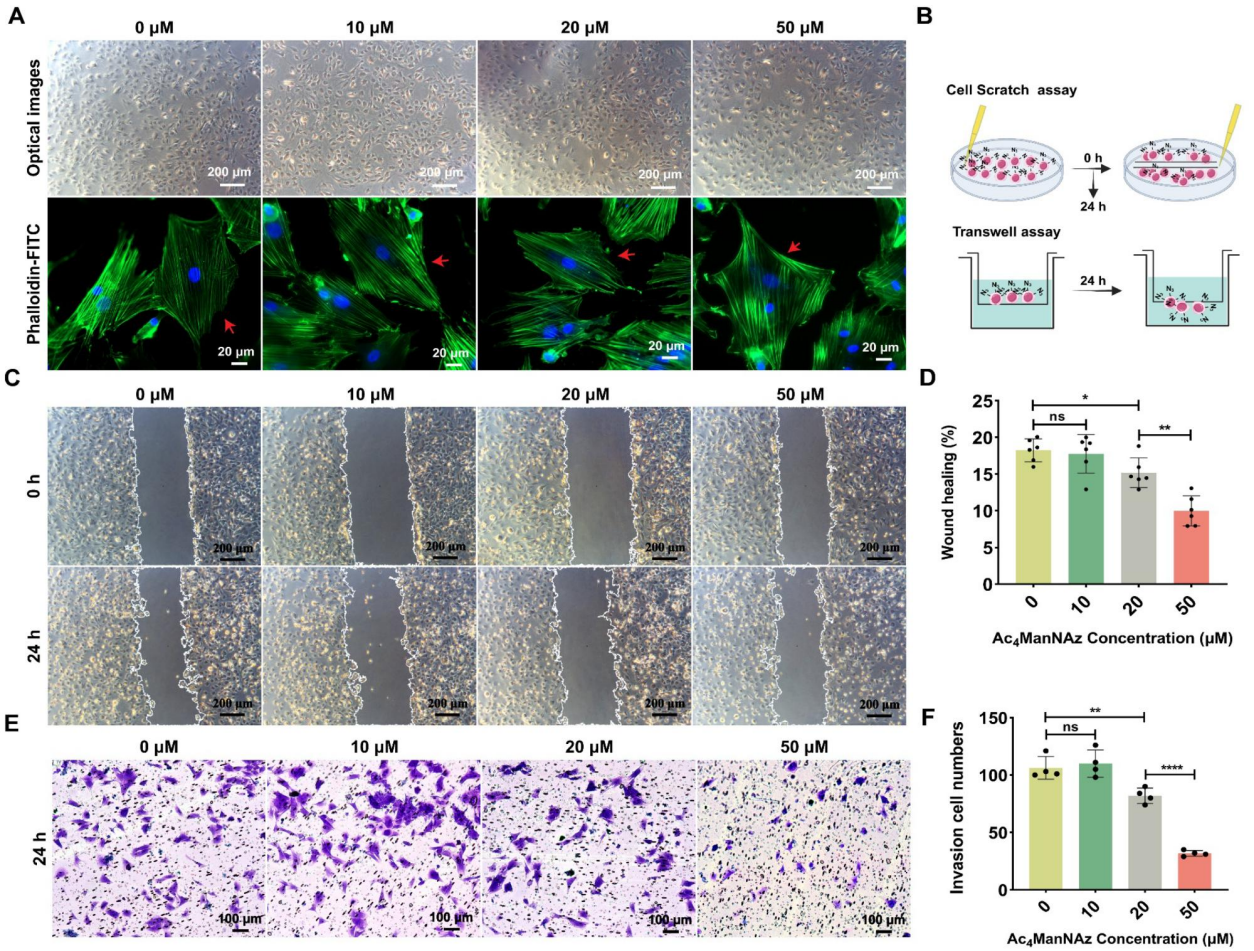
Adaptability of MSCs after treatment with Ac_4_ManNAz. (**A**) Representative images of MSCs after treatment with Ac_4_ManNAz by optical microscopic observation and phalloidin-FITC staining. The red arrow indicates the representative morphology of MSCs. Scale bar, 200 and 20 μm (bottom line). (**B**) A schematic illustration of migration assays. (**C**) Representative images of MSCs with various concentrations of Ac_4_ManNAz treatment by scratch assay. (**D**) Quantification of wound healing ratio by analyze the migration area (*n* = 6 from three biologically independent cultures). (**E**) Representative images of Ac_4_ManNAz-treated MSCs migrated through the trans-well membranes by crystal violet staining. Scale bar, 100 μm. (**F**) Quantitative analysis of the number of invasive cells (*n* = 4) from three biologically independent cultures. Data are expressed as mean ± SD; *P < 0.05, **P < 0.01, ****P < 0.0001 and ns represent *P* > 0.05.

Changes in morphology and migration enable MSCs to perform adaptive functions such as homing, differentiation, tissue repair and regeneration [45, 46]. To investigate the morphology and migratory capacity of Ac_4_ManNAz-treated MSCs, we examined the morphological changes in MSCs subjected to various concentrations of Ac_4_ManNAz using optical microscopy and phalloidin staining. The results showed that there were no significant morphology changes in Ac_4_ManNAz-treated MSCs, with cells maintaining their characteristic spread morphology on glass slides (Figure 2A). Next, migration and invasion capacities of Ac_4_ManNAz-treated MSCs were assessed by scratch and trans-well assays (Figure 2B). By assessing the distance of cell migration 24 h following the scratch, we confirmed that the migration ratios of MSCs were progressively decreased with escalating concentration of Ac_4_ManNAz treatment, especially in the 50-μM Ac_4_ManNAz-treated group (Figure 2C). The wound healing ratio was 18.25 ± 1.56% (0 μM), 17.77 ± 2.63% (10 μM), 15.19 ± 2.03% (20 μM) and 9.99 ± 2.04% (50 μM) (Figure 2D). Additionally, the invasion ability consisted of migration results, showing a significant reduction in invasion capacity in the 50-μM Ac_4_ManNAz-treated group compared to the 20-μM treatment group (Figure 2E and F). In particular, these findings indicate that Ac_4_ManNAz influences the migration and invasion capabilities of MSCs in a concentration-dependent manner. Research shows that different concentrations of Ac_4_ManNAz similarly affect the motility of A549 cancer cells and hUCB-EPCs [22, 23]. Given the intrinsic migratory properties of stem cells, they naturally migrate toward sites of injury or specific tumor locations. When employing bio-orthogonal technology for the surface modification of MSCs aimed at drug packaging in therapeutic applications, it is crucial to take into account the concentration of the metabolic labeling agent. Our study revealed that treatment with 50-μM Ac_4_ManNAz adversely affected the migratory capacity of MSCs. Consequently, when harnessing the homing properties of MSCs for therapeutic interventions, we recommend utilizing an Ac_4_ManNAz modification concentration below 50 μM to minimize the impact of metabolic monosaccharide concentrations on MSCs’ homing ability. Regardless of their homing capabilities, the functions of MSCs are remarkably multifaceted. Considering that treatment with 50-μM Ac_4_ManNAz significantly impacted the cellular motility of MSCs, we hypothesized that this specific concentration of azido-sugar would induce alterations in the physiological and biological functions of MSCs. Thus, we compared the functional changes in MSCs under 10- and 50-μM Ac_4_ManNAz treatment conditions to initially assess the effects of azido-sugar concentration on MSC biological function.

### Assessment of the biochemical function of MSC after treatment with Ac_4_ManNAz

**Figure 3:**
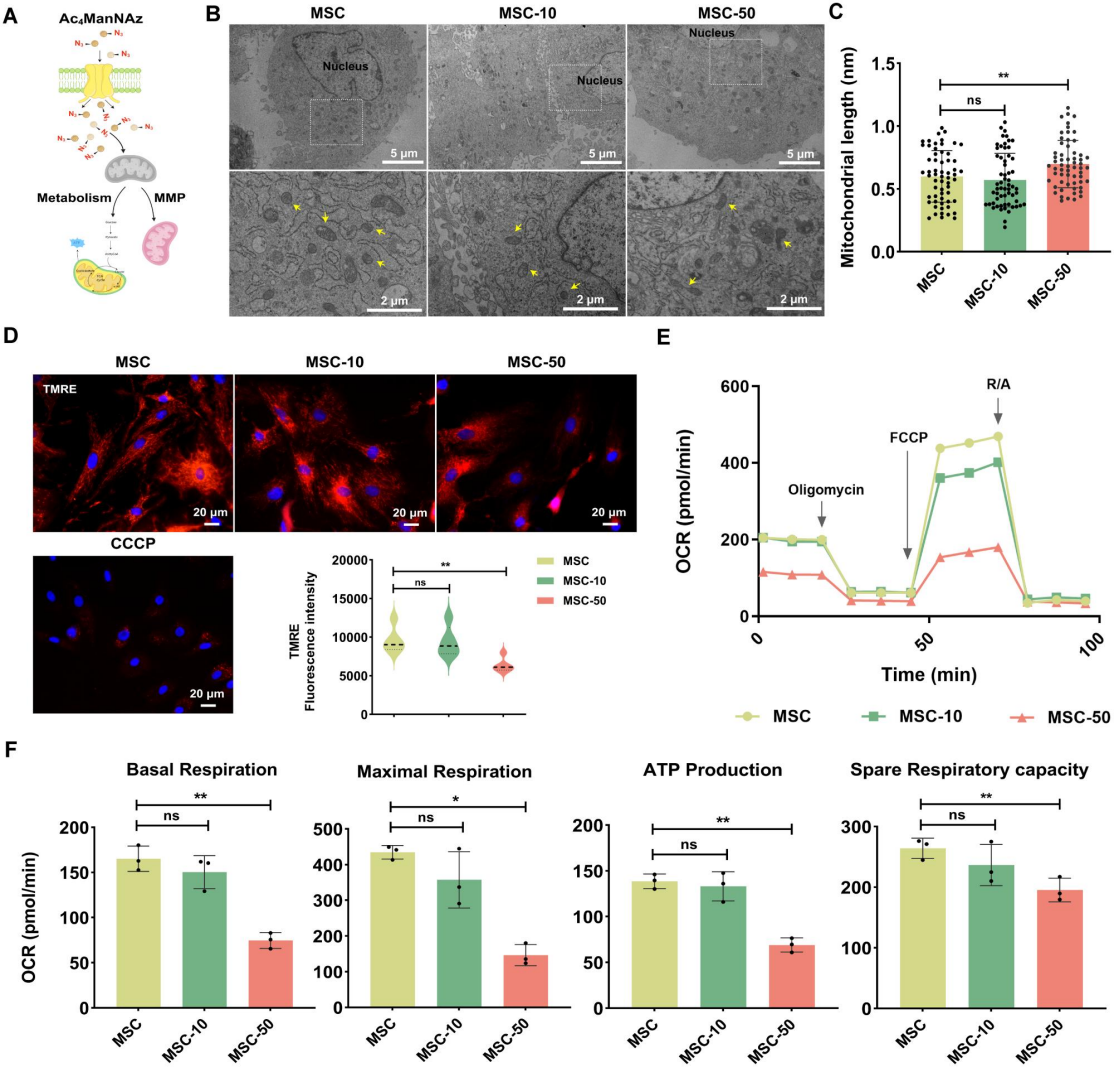
Analysis of mitochondrial functional index of MSCs following treatment with Ac_4_ManNAz. (**A**) a schematic illustration of mitochondrial functional analysis. (**B**) Representative mitochondrial TEM images of MSC and N_3_-MSC. Scale bar, 5 and 2 μm (magnification). The yellow arrows represent the mitochondrial morphology within the cytoplasm. (**C**) Quantitative analysis of the mitochondrial length (*n* = 3). (**D**) TMRE assay was used to detect MMP, and the fluorescence intensity of TMRE was quantitatively analyzed (CCCP represents the positive control). scale bar, 20 μm. (**E**) A representative graph of OCR outputs from Seahorse XFe24 Analyzer of MSCs and N_3_-MSCs (MSCs treated with 10- or 50-μM Ac_4_ManNAz). (**F**) Metabolic indicators were analyzed by quantifying the OCR (*n* = 3). Data are expressed as mean ± SD; *P < 0.05, **P < 0.01 and ns represent *P* > 0.05.

Mitochondria serve as both central metabolic hubs and key regulators of the cell, governing signal transduction, proliferation, differentiation and ionic homeostasis [47–49]. Glycosylation of proteins has been observed in mitochondria, where it plays a crucial role in the regulation of mitochondrial functions [50]. In light of this background, we assessed the effects of Ac_4_ManNAz on mitochondrial homeostasis. TEM images revealed changes in mitochondrial morphology in MSCs following treatment with Ac_4_ManNAz. In the control group (untreated MSC) and the MSC-10 group (MSCs treated with 10-μM Ac_4_ManNAz), mitochondria maintained oval or round shapes with distinct cristae structures (Figure 3A). Notably, the MSC-50 group (MSCs treated with 50-μM Ac_4_ManNAz) exhibited a partially elongated mitochondrion, and quantitative analysis confirmed a significant increase in mitochondrial length (Figure 3B and C). Given that mitochondria dynamically remodel their morphology through fusion and fission events to adapt to physiological demands, we analyzed mRNA expression of key regulators, including mitofusin 2 (*Mfn2*, outer membrane fusion marker) and dynamin-related protein 1 (*Drp1*, fission mediator). While *Mfn2* expression remained unchanged across groups, *Drp1* mRNA expression was significantly downregulated in azido-sugar-treated cells, specifically in the MSC-50 group (Supplementary Figure S2). This transcriptional suppression likely explains the observed elongation phenotype in the MSC-50 group [51].

Next, we assessed mitochondrial membrane potential using TMRE and JC-1 fluorescent probes. In the MSC-50 group, MMP was significantly depolarized, as indicated by a marked decrease in TMRE fluorescence intensity (Figure 3D) and a corresponding increase in green JC-1 monomer fluorescence within the mitochondrial matrix (Supplementary Figure S3A). Quantitative analysis confirmed a drastic reduction in the red/green fluorescence ratio, which fell to ∼3% in this group. In contrast, the MSC-10 group exhibited no significant alteration in MMP compared to the control group (Supplementary Figure S3B). To further determine the impact of azido-sugar on the bioenergetic profiles of MSCs, we evaluated the OCR. The results showed that OCR was decreased in the MSC-50 group (Figure 3E), consistent with the MMP analysis. As a result, basal respiration, maximal respiration, ATP production, and spare respiration capacity in the MSC-50 group were lower than those in the MSC group (Figure 3F). Indeed, the regulation of cellular metabolic functions is complex, involving cell-type specificity, spatiotemporal specificity and bidirectional interactions [52, 53]. A more profound investigation into the regulatory mechanisms governing the influence of 50-μM Ac_4_ManNAz on the biochemical properties of MSCs is imperative to substantiate its potential application.

### Immunoregulatory properties of Ac_4_ManNAz-treated MSCs

**Figure 4:**
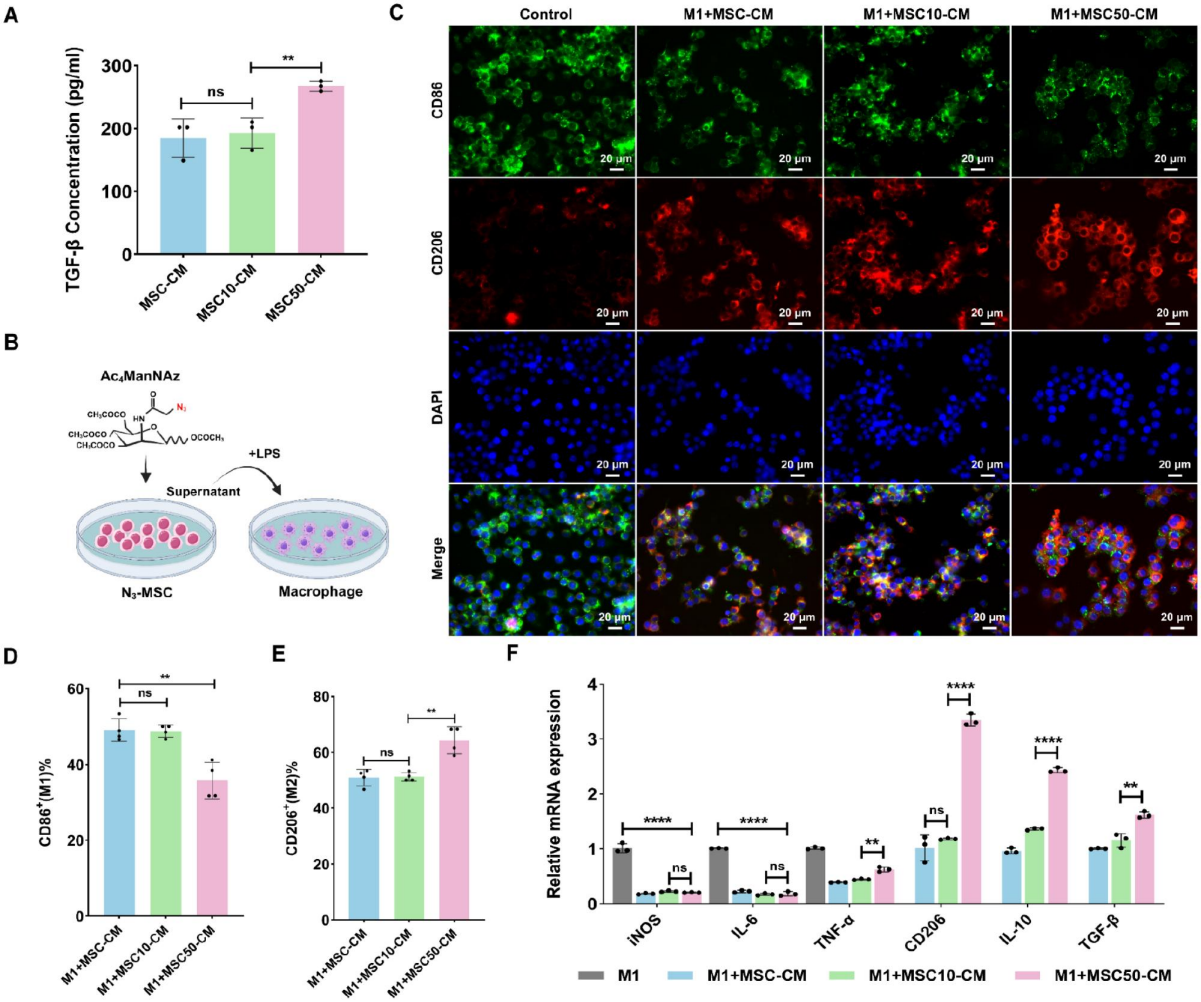
Immunoregulatory property of Ac_4_ManNAz-treated MSCs on M1 macrophages. (**A**) TGF-β protein secretion in the supernatant from MSC and Ac_4_ManNAz-treated MSCs (*n* = 3). (**B**) A schematic illustration of the co-culture assay. (**C**) IF staining of CD86^+^ macrophages and CD206^+^ macrophages in LPS-induced macrophages co-cultured with MSC-CM and N_3_-MSC-CM. Scale bar, 20 μm. (**D**) Quantification of CD86^+^(M1) macrophages percentage (*n* = 4). (**E**) Quantification of CD206^+^ (M2) macrophage percentage (*n* = 4). (**F**) qPCR analysis of marker genes of M1, M2 macrophages, anti-inflammatory, and pro-inflammatory cytokines across experimental groups (*n* = 3). Data are expressed as mean ± SD; *P < 0.05, **P < 0.01, ***P < 0.001, ****P < 0.0001 and ns represents *P* > 0.05.

Both direct cell–cell interactions and secreted factors are essential for MSCs to modulate macrophage activity. Contact with pro-inflammatory macrophages boosts tumor necrosis factor-stimulated gene-6 production and upregulates CD200 expression on MSCs [54]. Additionally, the paracrine cytokines released by MSCs mainly include TGF-β, prostaglandin E2 (PGE2), cyclooxygenase and indoleamine 2,3-dioxygenase [55, 56]. In the direct cell-to-cell co-culture system, we did not observe significant differences in *IL-6* and *IL-10* gene expression in M1 macrophages after co-culturing with MSC-50 compared with MSC. Furthermore, there were no notable alterations in *CD200* gene expression levels (Supplementary Figure S4). Subsequently, the analysis concentrated on the paracrine inflammatory cytokines produced by MSCs following treatment with Ac_4_ManNAz. Interestingly, soluble factors including activated TGF-β and IGF-2 expression in the cell supernatant were analyzed by ELISA assay. Interestingly, we found that activated TGF-β and IGF-2 protein levels were significantly upregulated in the 50-μM Ac_4_ManNAz-treated MSC-CM (MSC50-CM) group compared to the 10 μM Ac_4_ManNAz-treated MSC-CM (MSC10-CM) group (Figure 4A, Supplementary Figure S5A). We further examined the mRNA expression levels of additional bioactive factors in Ac_4_ManNAz-treated MSCs. The result showed that there was no significant difference in the gene expression levels of IGF-1 and hepatocyte growth factor (HGF) between the MSC-10 and MSC-50 groups. PGE2 gene expression level was significantly increased in the MSC-50 group. In contrast, vascular endothelial growth factor (VEGF) exhibited a decreasing trend in the MSC-50 group (Supplementary Figure S5B). The above results suggest that the Ac_4_ManNAz treatment appears to have a relatively substantial influence on the immune regulatory molecules of MSCs. To further assess the regulatory capacity of MSCs treated with Ac_4_ManNAz on macrophages, we co-cultured the RAW264.7 with the supernatant of Ac_4_ManNAz-treated MSCs under LPS conditions for 24 h (Figure 4B). IF staining was conducted to identify the M1 macrophage marker CD86 and the M2 macrophage marker CD206 (Figure 4C). Macrophages co-cultured with MSC50-CM resulted in a significantly decreased expression of M1 macrophages (Figure 4D) but a higher proportion of M2 phenotype macrophages compared to both the M1+MSC-CM and M1+MSC10-CM groups. ImageJ software analysis revealed that CD206^+^ macrophages constituted ∼64.25% of the total macrophages in the M1+MSC50-CM group, significantly higher than the 51.2% observed in the M1+MSC10-CM group (Figure 4E). Moreover, analysis of inflammatory cytokine mRNA expression levels showed that the M1+MSC50-CM group exhibited significant upregulation of anti-inflammatory (such as IL-10 and TGF-β1) compared to the M1+MSC10-CM group (Figure 4F). These results suggest that treatment with 50-μM Ac_4_ManNAz mainly influences the immunoregulatory properties of MSCs. The plasticity of MSCs is intricately linked to their surrounding environment. In this study, we observed significant alterations in the immunoregulatory functions of MSCs under conditions treated with 50-µM concentration of Ac_4_ManNAz. Notably, the expression of the HGF gene remained unaffected, while a downregulation trend was observed for the VEGF gene expression. Although these findings may initially appear to present contradictory functionalities within MSCs, they actually support the notion that MSC immunoregulation and pro-angiogenic activities are regulated by distinct signaling pathways [57]. This intelligent and differentiated response mechanism induced by monosaccharide metabolism provides a novel theoretical foundation for utilizing MSCs as an efficient and controllable cellular therapeutic agent.

### Mechanism of TGF-β upregulation in Ac_4_ManNAz-treated MSCs

**Figure 5:**
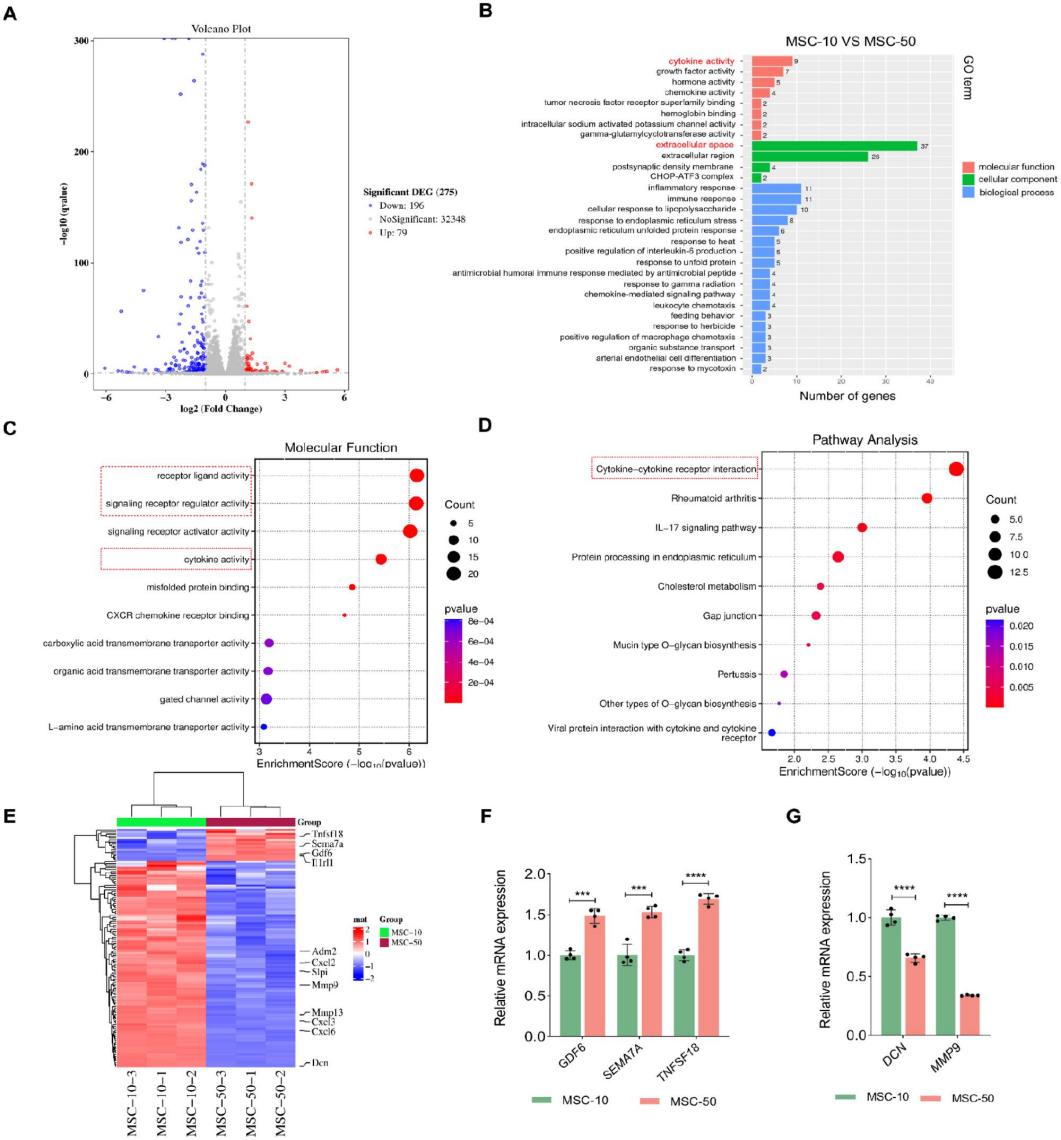
Transcriptomic analysis of Ac_4_ManNAz-treated MSCs. (**A**) Volcano plot of differentially expressed genes between MSC-10 and MSC-50. (**B**) GO annotations analysis focuses on molecular function, cellular component and biological process between MSC-10 and MSC-50. (**C**) GO enrichment analysis targeting cytokine activity, signaling receptor activator activity and signaling receptor regulator activity. (**D**) KEGG pathway enrichment analysis for cytokine-cytokine receptor interaction signaling pathway. (**E**) Heat map of gene expression related to cytokines and the extracellular space component of interest across experimental groups. (**F** and **G**) Quantitative qPCR analysis of genes associated with TGF-β signaling pathways (*n* = 4). Data are expressed as mean ± SD; ***P < 0.001 and ****P < 0.0001.

To explore the mechanism of metabolic labeling effects of 50-μM Ac_4_ManNAz on MSCs, we performed transcriptome analysis of MSCs treated with10- or 50-μM Ac_4_ManNAz, and untreated MSCs. mRNA extracted from each group was sequenced using the Illumina HiSeq platform. The number of differentially expressed genes (DEGs) differed significantly in the MSC-50 group compared to the MSC and MSC-10 groups (Supplementary Figure S6A). Venn diagrams revealed diverse shared genes among groups (Supplementary Figure S6B), with minimal DEGs between the MSC and MSC-10 groups. Subsequent analysis focused on gene expression changes in the MSC-50 group compared to the MSC-10 group. Volcano plot identified 79 upregulated and 196 downregulated genes in the MSC-50 group relative to the MSC-10 group (Figure 5A). Gene Ontology (GO) enrichment analysis of cellular component (CC), molecular function (MF) and biological process (BP) demonstrated that MSC-50-induced substantial alterations in extracellular space-related genes (in CC), cytokine activity (in MF) and inflammatory response (in BP) (Figure 5B). MF-associated genes primarily participated in receptor ligand activity (e.g. *SEMA7A, ADM2, SECTM1B* and *GDF6*), signaling receptor regulator activity and cytokine activity (e.g. *CXCL6, CXCL3, CXCL2* and *TNFS18*) (Figure 5C). The Kyoto Encyclopedia of Genes and Genomes (KEGG) annotation indicated that cytokine-cytokine receptor interaction was the primary differential signaling pathway between MSC-50 and MSC-10 (Figure 5D). Heatmap confirmed significant enrichment of cytokine-cytokine receptor signaling and membrane component gene sets (Figure 5E). Surprisingly, we found that cytokines receptor/glycoprotein-associated genes (*GDF6, SEMA7A, TNFSF18*) were upregulated in the MSC-50 group (Figure 5F), while extracellular space-related genes (*DCN* and *MMP9*) were significantly downregulated (Figure 5G). ECM molecules, particularly proteoglycans, show cooperative regulatory roles with cytokines and may possess intrinsic cytokine activity [58, 59]. Specifically, the upregulation of growth differentiation factor 6 (*GDF6,* associated with TGF-β function) [60], semaphorin7A (*SEMA7A*) [61] and downregulation of decorin (*DCN*, a negative regulator of TGF-β) [62] genes indicate the activated TGF-β signaling pathway in the MSC-50 group. Despite not detecting differential changes in *TGF-β* gene expression. However, RNA-seq results show activation of the TGF-β signaling pathway. In combination with the upregulated expression of TGF-β in the MSC50-CM group, it can be inferred that increased active TGF-β expression occurs at the post-translational level following 50 μM Ac_4_ManNAz treatment.

### ECM remodeling enhances active TGF-β secretion in Ac_4_ManNAz-treated MSCs

**Figure 6:**
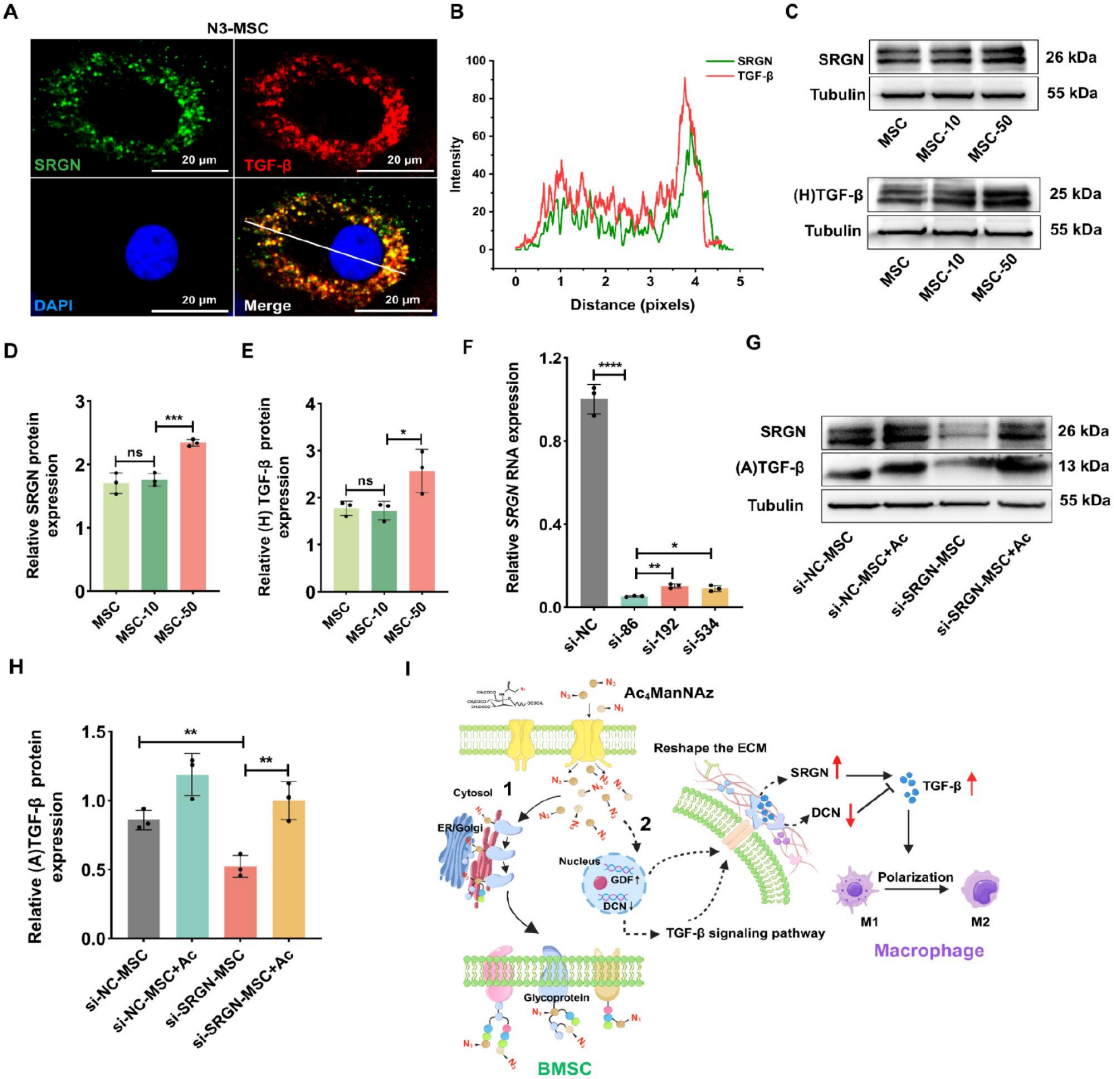
Analysis of the interaction between SRGN protein and TGF-β in N_3_-MSCs. (**A**) The IF staining of SRGN and TGF-β in the MSC-50 group. Scale bar, 20 μm. (**B**) Plot of pixel intensity along the white line in (**A**). (**C**) Western blot assessment of SRGN and TGF-β homodimer ((H) TGF-β) protein expression. (**D**) The gray value statistical analysis of SRGN proteins (*n* = 3). (**E**) The gray value statistical analysis of TGF-β proteins (*n* = 3). (**F**) mRNA expression of *SRGN* in MSCs before and after si-SRGN treatment (*n* = 3). (**G**) The rescue assay was performed in SRGN-knocked down MSCs by adding 50-µM Ac_4_ManNAz (Ac). (A)TGF-β represents the mature form of TGF-β protein. (**H**) The protein expression of SRGN was analyzed across the experimental groups (*n* = 3). (**I**) Schematic illustration of the mechanism by which the azido monosaccharide metabolic pathway (route 1) and treatment with 50 μM Ac_4_ManNAz modulate the immunological functions of MSCs (route 2). The diagram materials in this article are sourced from CNS knowall.com, provided by Shanghai Sennais (CNS) Biopharmaceutical Technology Co., Ltd. All rights and interpretations belong to the authors. Data are expressed as mean ± SD; *P < 0.05, **P < 0.01, ***P < 0.001, ****P < 0.0001 and ns represent *P* > 0.05.

The ECM provides mechanical support for cells and regulates their functions by interacting with growth factors, signal receptors and adhesion molecules [63, 64]. Our transcriptomic analysis revealed a reduction in the expression of *DCN* genes within the MSC-50 group, which may mitigate the inhibitory effects of TGF-β. Moreover, as an ECM glycoprotein, SRGN is essential for the storage, transport, secretion and protection of chemokines and bioactive molecules [65]. Although there was no significant difference in the expression of the *SRGN* gene, it has been reported that there is an interaction between SRGN and TGF-β proteins [66]. Consequently, we propose that the change in SRGN protein expression in the MSC-50 group may also exert an influence on the expression of TGF-β proteins. To validate this hypothesis, we employed the IF staining to assay the expression of SRGN and TGF-β proteins. Colocalization analysis revealed potential intracellular interaction between SRGN and TGF-β protein in the MSC-50 group, indicated by yellow overlap (Figure 6A and B). WB assay indicated the upregulated expression of SRGN and TGF-β homodimer proteins in the MSC-50 group (Figure 6C). The gray value statistical analysis shows that the SRGN protein expression in the MSC-50 group is 1.3-fold change higher than in the MSC-10 group (Figure 6D), while TGF-β protein expression is 1.5-fold change higher in the same comparison (Figure 6E). To further validate the interaction between SRGN and TGF-β in the MSC-50 group, si-RNA mediated SRGN knockdown assay was conducted in MSCs. In comparison to the control group, SRGN-siRNA-86 exhibited the highest silencing efficiency and was utilized in the following experiment (Figure 6F). Unsurprisingly, *TGF-β* mRNA expression was decreased following *SRGN* knockdown (Supplementary Figure S7A). We implemented a 50-µM Ac_4_ManNAz treatment as a corrective strategy to elucidate the expression profiles of SRGN and activated TGF-β proteins in MSCs before and after SRGN interference (Figure 6G). WB results indicated that si-SRGN effectively downregulated SRGN protein expression, which could be rescued by adding 50-µM Ac_4_ManNAz (Supplementary Figure S7B). In addition, SRGN-silenced MSCs showed decreased TGF-β protein expression to 1.6-fold change compared to the untreated MSC group. Interestingly, 50-µM Ac_4_ManNAz treatment restored TGF-β protein expression to levels similar to the untreated group (Figure 6H). This further confirms that SRGN is an essential ‘guardian’ for maintaining the homeostasis of TGF-β protein. Collectively, we propose a model for 50-µM Ac_4_ManNAz in regulating MSC functions: azido-sugar remodels the ECM of MSCs. This process ‘releases the brake’ by downregulating DCN. Additionally, ‘activates and stabilizes’ TGF-β via SRGN. The combined effect increases activated TGF-β secretion, promoting the polarization of M1 macrophages into M2 macrophages (Figure 6I).

### Impact of Ac_4_ManNAz on the differentiation potential of MSCs

To investigate the effects of different concentrations of Ac_4_ManNAz on the differentiation capacity of MSCs, the tri-lineage differentiation ability of MSCs were examined. We performed qPCR analysis of MSC differentiation markers at 7 days post-induction. Additionally, lineage-specific staining was conducted on cells differentiated along specific lineages at Day 18. The results indicated that for adipogenic differentiation, MSCs treated with 50-μM Ac_4_ManNAz retained their adipogenic differentiation potential. At Day 7, no significant differences were observed in the expression of adipogenic marker PPARγ among the groups, while increased expression of marker C/EBPα was observed in the MSC-50 group (Supplementary Figure S8A). Furthermore, Oil Red O staining at 18 days post-induction revealed that MSCs could be successfully induced into adipocytes both with and without azide-monosaccharide treatment (Supplementary Figure S8B). Quantitative analysis of lipid droplet area showed no statistically significant differences among the groups (Supplementary Figure S8C). For chondrogenic differentiation, Ac_4_ManNAz treatment did not affect the chondrogenic potential of MSCs. At Day 7, the expression of the chondrogenic marker Sox9 showed no significant statistical difference between groups (19% upregulation relative to GAPDH) (Supplementary Figure S9A). Under chondrogenic culture conditions, both MSCs and N_3_-MSCs displayed cartilaginous phenotypes and exhibited positive Alcian Blue staining by Day 18 (Supplementary Figure S9B). For osteogenic differentiation, we found that MSCs treated with 50-μM Ac_4_ManNAz exhibited reduced expression of the osteogenic marker gene Runx2 at Day 7 of induction (Supplementary Figure S10A). Furthermore, at Day 18 of induction, ARS staining revealed a significantly decreased area of calcium nodules in the MSC-50 group (Supplementary Figure S10B and C), indicating that 50-μM Ac_4_ManNAz treatment impaired the osteogenic differentiation capacity of MSCs. Notably, previous studies have demonstrated that alterations in mitochondrial function can affect the osteogenic potential of MSCs, and our findings are consistent with these prior reports [67].

Collectively, by evaluating the effects of different concentrations of Ac_4_ManNAz on MSC biological functions, we observed several intriguing findings, including impaired migration and osteogenic differentiation capacity but improved immunoregulatory capacities of MSCs. These discoveries extend beyond the traditional application of Ac_4_ManNAz solely as a metabolic marker for cells. It suggests that the functional state of MSCs may involve an ‘energy or resource allocation’ balance, where tilting toward paracrine immunomodulatory functions may come at the cost of partially compromising energy-consuming differentiation programs. Such MSCs may possess unique advantages for treating diseases where immunomodulation is the primary need and bone regeneration is secondary (such as autoimmune disorders and graft-versus-host disease). Conversely, they may be less suitable for scenarios requiring robust osteogenesis, such as critical bone defect repair. These mechanistic insights offer a novel framework for understanding MSC functional plasticity in the context of metabolic glycoengineering, providing a rationale for tailoring MSC properties for specific therapeutic applications.

## Conclusions

In summary, we systematically screen the effects of Ac_4_ManNAz at varying concentrations on MSC functions, providing new insights into metabolic labeling technology for clinical applications. We propose that the Ac_4_ManNAz concentration for MSC labeling should be comprehensively considered based on specific application requirements. The concentration of Ac_4_ManNAz is considered to be safely maintained within a range of 10–50 µM for MSC labeling. Particularly, treatment with 50-μM Ac_4_ManNAz resulted in a decreased migratory capability of MSCs but enhanced their immunoregulatory capacity on M1 macrophages. Furthermore, the mechanisms underlying the enhanced immunosuppressive abilities of MSCs following treatment with 50-μM Ac_4_ManNAz have been investigated, revealing alterations in ECM components that regulate the release of immune regulatory factors. Overall, our findings present a promising avenue for harnessing MSCs in immunotherapy and metrology through the innovative application of metabolic labeling technology.

## Supplementary Material

rbag044_Supplementary_Data

## Data Availability

Transcriptomic sequencing data are accessible on https://ngdc.cncb.ac.cn/. The GSA number is CRA028970. Other datasets from the current study are available from the corresponding author on reasonable request.

## References

[1] Jovic D , Yu Y, Wang D, Wang K, Li H, Xu F, Liu C, Liu J, Luo Y. A brief overview of global trends in MSC-based cell therapy. Stem Cell Rev Rep 2022;18:1525–45. 35344199 10.1007/s12015-022-10369-1 PMC8958818

[2] Kadri N , Amu S, Iacobaeus E, Boberg E, Le Blanc K. Current perspectives on mesenchymal stromal cell therapy for graft versus host disease. Cell Mol Immunol 2023;20:613–25. 37165014 10.1038/s41423-023-01022-z PMC10229573

[3] Wang ZX , Zhang XX, Xue LM, Wang GW, Li XD, Chen JW, Xu RX, Xu T. A controllable gelatin-based microcarriers fabrication system for the whole procedures of MSCs amplification and tissue engineering. Regen Biomater 2023;10:rbad068. 37638061 10.1093/rb/rbad068 PMC10458456

[4] Lee OJ , Keating A. Mesenchymal stromal cells: an update. Curr Opin Hematol 2025;32:270–8. 40668265 10.1097/MOH.0000000000000887

[5] Matthay MA , Calfee CS, Zhuo H, Thompson BT, Wilson JG, Levitt JE, Rogers AJ, Gotts JE, Wiener-Kronish JP, Bajwa EK, Donahoe MP, McVerry BJ, Ortiz LA, Exline M, Christman JW, Abbott J, Delucchi KL, Caballero L, McMillan M, McKenna DH, Liu KD. Treatment with allogeneic mesenchymal stromal cells for moderate to severe acute respiratory distress syndrome (START study): a randomised phase 2a safety trial. Lancet Respir Med 2019;7:154–62. 30455077 10.1016/S2213-2600(18)30418-1 PMC7597675

[6] Levy O , Kuai R, Siren EMJ, Bhere D, Milton Y, Nissar N, De Biasio M, Heinelt M, Reeve B, Abdi R, Alturki M, Fallatah M, Almalik A, Alhasan AH, Shah K, Karp JM. Shattering barriers toward clinically meaningful MSC therapies. Sci Adv 2020; 6: eaba6884. 32832666 10.1126/sciadv.aba6884 PMC7439491

[7] Ocansey DKW , Pei B, Yan Y, Qian H, Zhang X, Xu W, Mao F. Improved therapeutics of modified mesenchymal stem cells: an update. J Transl Med 2020;18:42. 32000804 10.1186/s12967-020-02234-x PMC6993499

[8] Moeinabadi-Bidgoli K , Mazloomnejad R, Beheshti Maal A, Asadzadeh Aghdaei H, Kazem Arki M, Hossein-Khannazer N, Vosough M. Genetic modification and preconditioning strategies to enhance functionality of mesenchymal stromal cells: a clinical perspective. Expert Opin Biol Ther 2023;23:461–78. 37073114 10.1080/14712598.2023.2205017

[9] Wechsler ME , Rao VV, Borelli AN, Anseth KS. Engineering the MSC secretome: a hydrogel focused approach. Adv Healthc Mater 2021; 10: e2001948. 33594836 10.1002/adhm.202001948 PMC8035320

[10] Chapman MS , Campbell PJ. Lineage tracing of stem cells decades after blood and bone-marrow transplantation. Nature 2025;635:926–34. 10.1038/d41586-025-00345-3 39910360

[11] Mehrjoo M , Karkhaneh A, Nazarpak MH, Alishahi M, Bonakdar S. Hydroxyapatite-induced bioactive and cell-imprinted polydimethylsiloxane surface to accelerate osteoblast proliferation and differentiation: an study on preparation and differentiating capacity. Biomed Mater 2025;20:045024. 10.1088/1748-605X/ade5e0 40532725

[12] Layek B , Sadhukha T, Prabha S. Glycoengineered mesenchymal stem cells as an enabling platform for two-step targeting of solid tumors. Biomaterials 2016;88:97–109. 26946263 10.1016/j.biomaterials.2016.02.024

[13] Co CM , Izuagbe S, Zhou J, Zhou N, Sun XK, Borrelli J, Tang LP. Click chemistry-based pre-targeting cell delivery for cartilage regeneration. Regen Biomater 2021;8:rbab018. 34211730 10.1093/rb/rbab018 PMC8240595

[14] Helmeke MMB , Haynie-Cion RL, Pratt MR. Achieving cell-type selectivity in metabolic oligosaccharide engineering. RSC Chem Biol 2025;6:1506–20. 40843436 10.1039/d5cb00168d PMC12364120

[15] Shen L , Cai KM, Yu J, Cheng JJ. Novel liposomal azido mannosamine lipids on metabolic cell labeling and imaging via Cu-Free click chemistry. Bioconjug Chem 2019;30:2317–22. 31403278 10.1021/acs.bioconjchem.9b00509 PMC7470023

[16] Nagahama K , Kimura Y, Takemoto A. Living functional hydrogels generated by bioorthogonal cross-linking reactions of azide-modified cells with alkyne-modified polymers. Nat Commun 2018;9:2195. 29875358 10.1038/s41467-018-04699-3 PMC5989231

[17] Altmann S , Mut J, Wolf N, Meißner-Weigl J, Rudert M, Jakob F, Gutmann M, Lühmann T, Seibel J, Ebert R. Metabolic glycoengineering in hMSC-TERT as a model for skeletal precursors by using modified azide/alkyne monosaccharides. Int J Mol Sci 2021;22:2820. 33802220 10.3390/ijms22062820 PMC7999278

[18] Lu YT , Chen TY, Lin HH, Chen YW, Lin YX, Le DY, Huang YH, Wang AHJ, Lee CC, Ling TY. Small extracellular vesicles engineered using click chemistry to express chimeric antigen receptors show enhanced efficacy in acute liver failure. J Extracell Vesicles 2025;14:e70044. 39901768 10.1002/jev2.70044 PMC11791321

[19] Yun WS , Shim MK, Lim S, Song S, Kim J, Yang S, Hwang HS, Kim MR, Yoon HY, Lim DK, Sun IC, Kim K. Mesenchymal stem cell-mediated deep tumor delivery of gold nanorod for photothermal therapy. Nanomaterials (Basel) 2022;12:3410. 36234538 10.3390/nano12193410 PMC9565344

[20] Wratil PR , Horstkorte R, Reutter W. Metabolic glycoengineering with -acyl side chain modified mannosamines. Angew Chem Int Ed Engl 2016;55:9482–512. 27435524 10.1002/anie.201601123

[21] Conroy LR , Hawkinson TR, Young LEA, Gentry MS, Sun RC. Emerging roles of N-linked glycosylation in brain physiology and disorders. Trends Endocrinol Metab 2021;32:980–93. 34756776 10.1016/j.tem.2021.09.006 PMC8589112

[22] Han SS , Shim HE, Park SJ, Kim BC, Lee DE, Chung HM, Moon SH, Kang SW. Safety and optimization of metabolic labeling of endothelial progenitor cells for tracking. Sci Rep 2018;8:13212. 30181604 10.1038/s41598-018-31594-0 PMC6123424

[23] Han SS , Lee DE, Shim HE, Lee S, Jung T, Oh JH, Lee HA, Moon SH, Jeon J, Yoon S, Kim K, Kang SW. Physiological effects of Ac4ManNAz and optimization of metabolic labeling for cell tracking. Theranostics 2017;7:1164–76. 28435456 10.7150/thno.17711 PMC5399584

[24] Lu W , Allickson J. Mesenchymal stromal cell therapy: progress to date and future outlook. Mol Ther 2025;33:2679–88. 39916329 10.1016/j.ymthe.2025.02.003 PMC12172195

[25] Blanc KL , Dazzi F, English K, Farge D, Galipeau J, Horwitz EM, Kadri N, Krampera M, Lalu MM, Nolta J, Patel NM, Shi Y, Weiss DJ, Viswanathan S. ISCT MSC committee statement on the US FDA approval of allogenic bone-marrow mesenchymal stromal cells. Cytotherapy 2025;27:413–6. 39864015 10.1016/j.jcyt.2025.01.005

[26] Li J , Zhang CM, Li JY, Gao RJ, Yang MZ, Yu LK, Zhang W, Zhou GQ, Shen WZ, Zhang JC, Jia G, Ge K. Strontium-incorporated hydroxyapatite nanocomposites promoting bone formation and angiogenesis by modulating M2 macrophage polarization in the bone microenvironment. Regen Biomater 2025;12:rbaf066. 40799901 10.1093/rb/rbaf066 PMC12341688

[27] Shi ZJ , Chen SN, Cai WK, Chen W, Tang Y. Advances in photobiomodulation: effects on mesenchymal stem cells and their paracrine factors. Stem Cell Rev Rep 2025;21:1918–30. 40637970 10.1007/s12015-025-10934-4

[28] Zhou C , Zhang B, Yang Y, Jiang Q, Li T, Gong J, Tang H, Zhang Q. Stem cell-derived exosomes: emerging therapeutic opportunities for wound healing. Stem Cell Res Ther 2023;14:107. 37101197 10.1186/s13287-023-03345-0 PMC10134577

[29] Lotfy A , AboQuella NM, Wang H. Mesenchymal stromal/stem cell (MSC)-derived exosomes in clinical trials. Stem Cell Res Ther 2023;14:66. 37024925 10.1186/s13287-023-03287-7 PMC10079493

[30] Sun C , Su JW, Wang Z, Liu CJ, Yi XZY, Chen WM, Zhang D, Yu AX. Engineering stem cell exosomes promotes the survival of multi-territory perforator flap in diabetes via regulating anti-inflammatory and angiogenesis. Regen Biomater 2025;12:rbaf075. 40837703 10.1093/rb/rbaf075 PMC12364439

[31] Wu J , Li SQ, Wang H, Qi YB, Tao S, Tang PF, Liu DH. High-yield BMSC-derived exosomes by the 3D culture system to enhance the skin wound repair. Regen Biomater 2025;12:rbaf022. 40309353 10.1093/rb/rbaf022 PMC12041419

[32] Anselmi C , Soares IPM, Chang SR, Cardoso LM, de Carvalho ABG, Dal-Fabbro R, Costa CAD, Bottino MC, Hebling J. Quercetin-calcium hydroxide scaffolds modulate dental pulp stem cell response in vitro under a simulated inflammatory environment. Int Endod J 2025;58:1073–90. 40285990 10.1111/iej.14243 PMC12162201

[33] Du L , Lin L, Li Q, Liu K, Huang Y, Wang X, Cao K, Chen X, Cao W, Li F, Shao C, Wang Y, Shi Y. IGF-2 preprograms maturing macrophages to acquire oxidative phosphorylation-dependent anti-inflammatory properties. Cell Metab 2019;29:1363–75 e8. 30745181 10.1016/j.cmet.2019.01.006

[34] Cai GL , Zhao WK, Zhu TH, Oliveira AL, Yao X, Zhang YP. Effects of protein conformational transition accompanied with crosslinking density cues in silk fibroin hydrogels on the proliferation and chondrogenesis of encapsulated stem cells. Regen Biomater 2025;12:rbaf019. 40290449 10.1093/rb/rbaf019 PMC12033033

[35] Yang C , Cai WB, Xiang P, Liu Y, Xu H, Zhang W, Han FX, Luo ZP, Liang T. Viscoelastic hydrogel combined with dynamic compression promotes osteogenic differentiation of bone marrow mesenchymal stem cells and bone repair in rats. Regen Biomater 2025;12:rbae136. 39845143 10.1093/rb/rbae136 PMC11751691

[36] Sears V , Danaoui Y, Ghosh G. Impact of mesenchymal stem cell-secretome-loaded hydrogel on proliferative and migratory activities of hyperglycemic fibroblasts. Mater Today Commun 2021;27:102285. 33937466 10.1016/j.mtcomm.2021.102285 PMC8087264

[37] Zhuo Y , Li WS, Lu W, Li X, Ge LT, Huang Y, Gao QT, Deng YJ, Jiang XC, Lan ZW, Deng Q, Chen YH, Xiao Y, Lu S, Jiang F, Liu Z, Hu L, Liu Y, Ding Y, He ZW, Tan DA, Duan D, Lu M. TGF-β1 mediates hypoxia-preconditioned olfactory mucosa mesenchymal stem cells improved neural functional recovery in parkinson’s disease models and patients. Mil Med Res 2024;11:48. 39034405 10.1186/s40779-024-00550-7 PMC11265117

[38] Wieteska L , Taylor AB, Punch E, Coleman JA, Conway IO, Lin YF, Byeon CH, Hinck CS, Krzysiak T, Ishima R, Lopez-Casillas F, Cherepanov P, Bernard DJ, Hill CS, Hinck AP. Structures of TGF-beta with betaglycan and signaling receptors reveal mechanisms of complex assembly and signaling. Nat Commun 2025;16:1778. 40011426 10.1038/s41467-025-56796-9 PMC11865472

[39] Zelisko N , Lesyk R, Stoika R. Structure, unique biological properties, and mechanisms of action of transforming growth factor beta. Bioorg Chem 2024;150:107611. 38964148 10.1016/j.bioorg.2024.107611

[40] Hourani T , Sharma A, Luwor RB, Achuthan AA. Transforming growth factor-beta in tumor microenvironment: understanding its impact on monocytes and macrophages for its targeting. Int Rev Immunol 2025;44:82–97. 39377520 10.1080/08830185.2024.2411998

[41] Xu XC , Xiao L, Xu YM, Zhuo J, Yang X, Li L, Xiao NAQ, Tao J, Zhong Q, Li YF, Chen YL, Du ZB, Luo K. Vascularized bone regeneration accelerated by 3D-printed nanosilicate-functionalized polycaprolactone scaffold. Regen Biomater 2021;8:rbab061. 34858634 10.1093/rb/rbab061 PMC8633727

[42] Abbas OL , Özatik O, Gönen ZB, Koçman AE, Dağ I, Özatik FY, Bahar D, Musmul A. Bone marrow mesenchymal stem cell transplantation enhances nerve regeneration in a rat model of hindlimb replantation. Plast Reconstr Surg 2019;143:758e–68e. 10.1097/PRS.0000000000005412 30921125

[43] Mao D , Zhang C, Liu J, Wang X, Li B, Yan H, Hu F, Kong D, Wang Z, Liu B, Kenry. Bio-orthogonal click reaction-enabled highly specific cellularization of tissue engineering scaffolds. Biomaterials 2020;230:119615. 31776020 10.1016/j.biomaterials.2019.119615

[44] Battigelli A , Almeida B, Shukla S, Rocha AD, Shukla A. Inducing mesenchymal stem cell attachment on non-cell adhesive hydrogels through click chemistry. Chem Commun (Camb) 2020;56:7661–4. 32520061 10.1039/d0cc03403g

[45] Nitzsche F , Muller C, Lukomska B, Jolkkonen J, Deten A, Boltze J. Concise review: MSC adhesion Cascade-Insights into homing and transendothelial migration. Stem Cells 2017;35:1446–60. 28316123 10.1002/stem.2614

[46] Long XX , Dong YZ, Guo T, Zhang YT, Liu P, Wu YP, Lu H, Wang XW, Nie HM, Teoh SH, Wen F, Wang ZY. Mechanically reinforced core-shell scaffold with integrated structure and function for accelerated tendon repair. Regen Biomater 2025;12:rbaf088. 40979830 10.1093/rb/rbaf088 PMC12448295

[47] Picard M , Shirihai OS. Mitochondrial signal transduction. Cell Metab 2022;34:1620–53. 36323233 10.1016/j.cmet.2022.10.008 PMC9692202

[48] Chen W , Zhao H, Li Y. Mitochondrial dynamics in health and disease: mechanisms and potential targets. Signal Transduct Target Ther 2023;8:333. 37669960 10.1038/s41392-023-01547-9 PMC10480456

[49] Wang SZ , Liu JL, Zhou LX, Xu H, Zhang D, Zhang X, Wang Q, Zhou Q. Research progresses on mitochondrial-targeted biomaterials for bone defect repair. Regen Biomater 2024;11:rbae082. 39055307 10.1093/rb/rbae082 PMC11272180

[50] Kadam A , Jadiya P, Tomar D. Post-translational modifications and protein quality control of mitochondrial channels and transporters. Front Cell Dev Biol 2023;11:1196466. 37601094 10.3389/fcell.2023.1196466 PMC10434574

[51] Landoni JC , Kleele T, Winter J, Stepp W, Manley S. Mitochondrial structure, dynamics, and physiology light microscopy to disentangle the network. Annual Review of Cell and Developmental Biology 2024;40:219–40. 10.1146/annurev-cellbio-111822-114733 38976811

[52] Giacomello M , Pyakurel A, Glytsou C, Scorrano L. The cell biology of mitochondrial membrane dynamics. Nat Rev Mol Cell Biol 2020;21:204–24. 32071438 10.1038/s41580-020-0210-7

[53] Dong ZX , Han WJ, Jiang PY, Hao LJ, Fu XL. Regulation of mitochondrial network architecture and function in mesenchymal stem cells by micropatterned surfaces. Regen Biomater 2024;11:rbae052. 38854681 10.1093/rb/rbae052 PMC11162196

[54] Li Y , Zhang D, Xu L, Dong L, Zheng J, Lin Y, Huang J, Zhang Y, Tao Y, Zang X, Li D, Du M. Cell-cell contact with proinflammatory macrophages enhances the immunotherapeutic effect of mesenchymal stem cells in two abortion models. Cell Mol Immunol 2019;16:908–20. 30778166 10.1038/s41423-019-0204-6 PMC6884632

[55] Kulesza A , Paczek L, Burdzinska A. The role of COX-2 and PGE2 in the regulation of immunomodulation and other functions of mesenchymal stromal cells. Biomedicines 2023;11:445. 36830980 10.3390/biomedicines11020445 PMC9952951

[56] Hui YF , Jiao X, Yang L, Lu DJ, Han YB, Yang W, Cao YL, Miao YX, Gong SQ, Wei MJ. Indoleamine-2,3-dioxygenase: an important controller in maintaining mesenchymal stem cell-mediated immunomodulatory homeostasis. Acta Pharm Sin B 2025;15:3404–18. 40698141 10.1016/j.apsb.2025.04.022 PMC12278408

[57] Harrell CR , Fellabaum C, Jovicic N, Djonov V, Arsenijevic N, Volarevic V. Molecular mechanisms responsible for therapeutic potential of mesenchymal stem cell-derived secretome. Cells 2019;8:467. 31100966 10.3390/cells8050467 PMC6562906

[58] Pang QM , Chen ZL, Li XH, Zhan JD, Huang W, Lei YT, Bao W. Cytokine-activated mesenchymal-stem-cell-derived extracellular matrix facilitates cartilage repair by enhancing chondrocyte homeostasis and chondrogenesis of recruited stem cells. Research (Wash D C) 2025;8:0700. 41049612 10.34133/research.0700 PMC12494090

[59] Zheng YJ , Lei JD, Zhang A, Cao C, Xu A, Zhou MN, Lin FQ. Regulatory mechanisms of transforming growth factor-3 in senescence of fibroblast associated with refractory skin diseases. Exp Gerontol 2025;211:112900. 41015205 10.1016/j.exger.2025.112900

[60] Pan Q , Kay T, Depince A, Adolfi M, Schartl M, Guiguen Y, Herpin A. Evolution of master sex determiners: TGF-beta signalling pathways at regulatory crossroads. Philos Trans R Soc Lond B Biol Sci 2021;376:20200091. 34247498 10.1098/rstb.2020.0091 PMC8273507

[61] Peng H , Sun F, Jiang Y, Guo Z, Liu X, Zuo A, Lu D. Semaphorin 7a aggravates TGF-beta1-induced airway EMT through the FAK/ERK1/2 signaling pathway in asthma. Front Immunol 2023;14:1167605. 38022556 10.3389/fimmu.2023.1167605 PMC10646317

[62] Wang S , Qu Y, Fang X, Ding Q, Zhao H, Yu X, Xu T, Lu R, Jing S, Liu C, Wu H, Liu Y. Decorin: a potential therapeutic candidate for ligamentum flavum hypertrophy by antagonizing TGF-beta1. Exp Mol Med 2023;55:1413–23. 37394592 10.1038/s12276-023-01023-y PMC10394053

[63] Sui JC , Pragnere S, Kurniawan NA. Revisiting the biophysical aspects of extracellular-matrix-mimicking hydrogels: what cells see what cells feel. Biomater Sci 2025;13:5297–324. 40878170 10.1039/d5bm00210a PMC12394797

[64] Chen WH , Chen MS, Chen SY, Wang SR, Huang ZJ, Zhang LN, Wu JM, Peng WJ, Li HQ, Wen F. Decellularization of fish tissues for tissue engineering and regenerative medicine applications. Regen Biomater 2025;12:rbae138. 39776859 10.1093/rb/rbae138 PMC11703550

[65] Tellez-Gabriel M , Tekpli X, Reine TM, Hegge B, Nielsen SR, Chen M, Moi L, Normann LS, Busund L-TR, Calin GA, Mælandsmo GM, Perander M, Theocharis AD, Kolset SO, Knutsen E. Serglycin is involved in TGF-beta induced epithelial-mesenchymal transition and is highly expressed by immune cells in breast cancer tissue. Front Oncol 2022;12:868868. 35494005 10.3389/fonc.2022.868868 PMC9047906

[66] Zhang Z , Deng Y, Zheng G, Jia X, Xiong Y, Luo K, Qiu Q, Qiu N, Yin J, Lu M, Liu H, Gu Y, He Z. SRGN-TGFbeta2 regulatory loop confers invasion and metastasis in triple-negative breast cancer. Oncogenesis 2017; 6: e360. 28692037 10.1038/oncsis.2017.53 PMC5541705

[67] Li QQ , Gao ZW, Chen Y, Guan MX. The role of mitochondria in osteogenic, adipogenic and chondrogenic differentiation of mesenchymal stem cells. Protein Cell 2017;8:439–45. 28271444 10.1007/s13238-017-0385-7 PMC5445026

