# Supplementary material for: Beyond labeling: differential Ac4ManNAz dosing as a tool for functional manipulation of mesenchymal stem cells
Source: Regen Biomater. 2026 Mar 9;13:rbag044. doi: 10.1093/rb/rbag044 (PMC13138843; doi:10.1093/rb/rbag044)
Supplement: rbag044_Supplementary_Data [file rbag044_supplementary_data.zip › Supplementary Material - Proof.docx]

**Beyond Labeling: Differential Ac_4_ManNAz Dosing as a Tool for Functional Manipulation of Mesenchymal Stem Cells**

**Xueying Zhao^1^, Suqing Li^1^, Xingyu Jiang^1^, Yuyang Ma^1^, Weike Fan^1^, Luzhong Zhang^1*^, Xin Liu^2*^, Yumin Yang^1*^**

1. Medical School of Nantong University, Key laboratory of Neuroregeneration of Jiangsu and Ministry of Education, Co-innovation Center of Neuroregeneration, Nantong University, 226001 Nantong, P. R. China.

2. The Third Clinical Medical College, Affiliated Hospital of Integrated Traditional Chinese and Western Medicine, Nanjing University of Chinese Medicine, 210028, P.R. China

* Correspondence: [yangym@ntu.edu.cn](mailto:yangym@ntu.edu.cn), [liuxin@njucm.edu.cn](mailto:liuxin@njucm.edu.cn), [zhangluzhong@ntu.edu.cn](mailto:zhangluzhong@ntu.edu.cn)

**Supplementary Tables and Figures**

**Table S1:** qPCR primers

| **Primer Name** | **Sequence (5’-3’)** |
| --- | --- |
| GAPDH | F- CATCGTGGAAGGGCTCAT |
|  | R- CGCCACAGCTTTCCAGAG |
| CD29 | F- ATCATGCAGGTTGCAGTTTG |
|  | R- CGTGGAAAACACCAGCAGT |
| CD90 | F- CCACAAGCTCCAATAAAACTATCAA |
|  | R- AGCAGCCAGGAAGTGTTTTG |
| CD45 | F- ATTACCTGGAATCCCCCTCAAA |
|  | R- TTGTGAAATGACACATTGCAGC |
| CD11b | F- GGGAGCCCCACACTGATA |
|  | R- AGAGGGAGGCCCCAAAATA |
| INOS | F- GACGAGACGGATAGGCAGAG |
|  | R- CACATGCAA GGAAGGGAACT |
| CD206 | F- ATGGATTGCCCTGAACAGCA |
|  | R- CTCGTCAGCACCCCAGTTAG |
| IL-6 | F- TCCATCCAGTTGCCCTTCTTGG |
|  | R- CCACGATTTCCCAGAGAACTG |
| TNF-α | F- CCCCAAAGGGATGAGAAGTT |
|  | R- CACTTGGTGGTTTGCTACGA |
| IL-10 | F- CTGGACAACATACTGCTAAC |
|  | R- AAATGCTCCTTGATTTCTGG |
| TGF-β | F- CCTATTTAAGAACACCCACTTT |
|  | R- TCCTGAATAATTTGAGGTTGAG |
| GDF6 | F- TGCGGGCAATAATGTGGTCT |
|  | R- CAGTTAGGCCTTCCTCCGTG |
| SEMA7A | F- ATCTCCGCCGTCTGGAAAGG |
|  | R- AGGTAGACCTTGCCTCGTCC |
| TNFSF18 | F- CAAGCCAACTGCCAAAGAGC |
|  | R- TGAAGTATCTCCAGCATCCCA |
| DCN | F- TACAGGGGAGAAACCCAGACT |
|  | R- ATGGTCCAGCCCAAGAGACT |
| MMP9 | F- GATCCCCAGAGCGTTACTCG |
|  | R- GTTGTGGAAACTCACACGCC |
| SLIP | F- GTCTGCCTTCCCCCAGTGTA |
|  | R- CGGAACCGGGAAACCTGAAAG |
| MFN2 | F- CCCTTACCAGCTAGAAACGAGA |
|  | R- CCTCAGCCATGTGTCGCTTA |
| DRP-1 | F- AGGGACCTTCTTCCCAGAGG |
|  | R- CCATTCTTCTGCTTCAACTCCATT |
| PEG2 | F- ATGGTGTGCTGCGAGTGATGG |
|  | R- CAAGGCTGGATGTG TGAGTGTCG |
| IGF-1 | F- GACCCGGGACGTACCAAAAT |
|  | R- CGAGCTGGTAAAGGTGAGCA |
| IGF-2 | F- AGCGGCCTCCTTATCCAACT |
|  | R- CGTGTCAACAAGCTCCCCTC |
| HGF | F- CCGGCTTGCAACAGGATTCTT |
|  | R- GGACCGGCAGAAGTTTGGT |
| VEGF | F- TTGAGACCCTGGTGGACATC |
|  | R- CTCCAGGGCTTCATCATTGC |
| TGS-6 | F- ATGACGATGTCCACGGCTTT |
|  | R- GACGGACGCATCACTCAGAA |
| CD200 | F- GATGGGCAGTCCGGTATTCA |
|  | R- TGGGTCACCACTTCCACTTG |
| PPARγ | F- AAGGCTGCAGCGCTAAATTC |
|  | R- ATGGCATCTCTGTGTCAACCAT |
| C/EBPα | F- CCTCCGTCCCTGTCCTTAGA |
|  | R- AAGCAAGGGGCTAAGAACCC |
| Sox9 | F- CACAAGAAAGACCACCCCGA |
|  | R- ATGTGAGTCTGTTCGGTGGC |
| Runx2 | F- CACAAGTGCGGTGCAAACTT |
|  | R- ATGACTCGGTTGGTCTCGGT |
| Alp | F- TCTGAGCATGACCAACCAGG |
|  | R- TACCCCGAGATCCGTTCCTC |

**Table S2:** SRGN siRNA primers

| **Primer name** | **Sequence (5’-3’)** |
| --- | --- |
| SRGN (Norway rat): siRNA-192 | CGACCUAAUAGCAGAGGAATT |
|  | UUCCUCUGCUAUUAGGUCGTT |
| SRGN (Norway rat): siRNA-86 | GAUCUUCAGUUCAAGGUUATT |
|  | UAACCUUGAACUGAAGAUCTT |
| SRGN (Norway rat): siRNA-534 | CCAAGAACAACCAGGAGAUTT |
|  | AUCUCCUGGUUGUUCUUGGTT |
| Negative control | UUCUCCGAACGUGUCACGUTT |
|  | UUCUCCGAACGUGUCACGUTT |


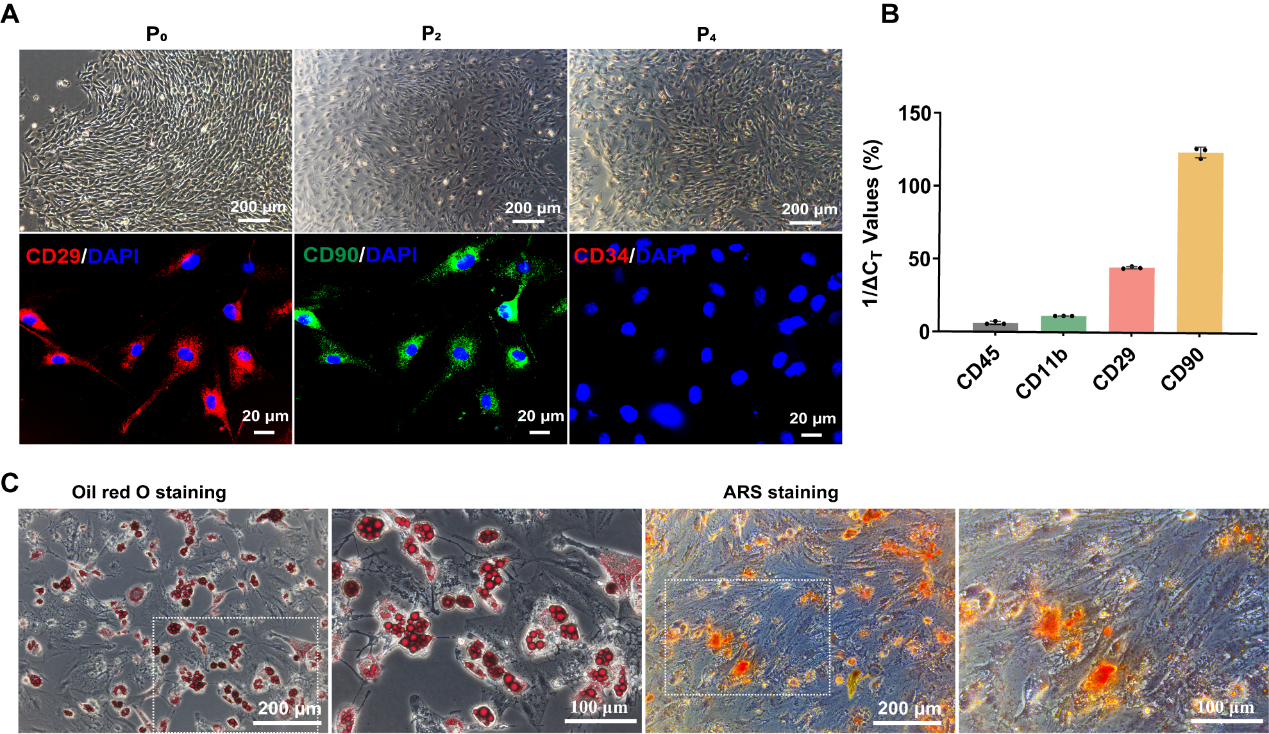


**Figure S1.** Characteristic of MSCs. (A) Representative optical microscope images of MSCs at passage 0, 2 and 4. IF staining of CD29, CD90 and CD34 in MSCs (bottom line). Scale bar, 200, and 20 μm (magnification). (B) mRNA expression of CD markers in MSCs (n = 3). (C) The adipogenic and osteogenic differentiation potential of MSCs. Cells were stained with Oil red O and ARS staining after cultured in differentiation medium for 14 days. Scale bar, 200, and 100 μm (magnification).


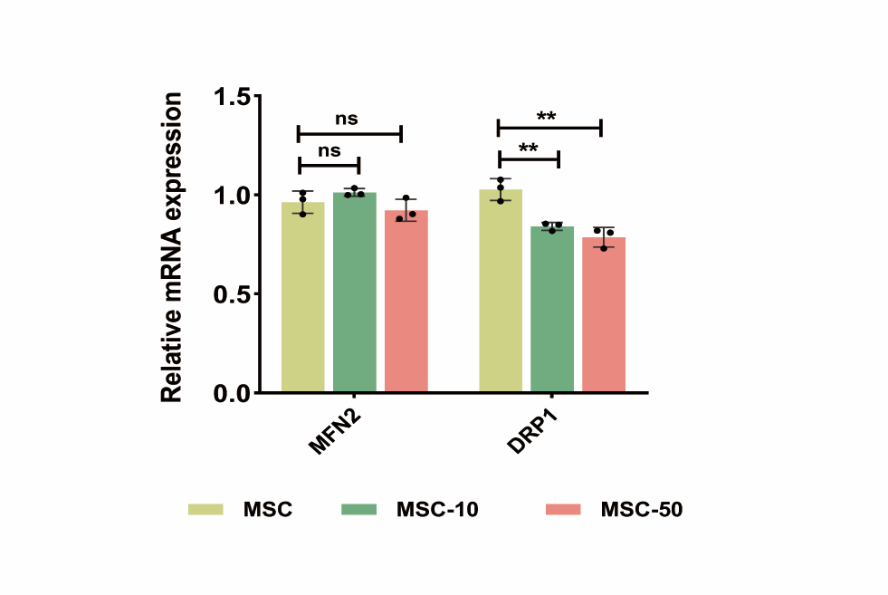


**Figure S2.** mRNA levels of fusion/fission genes (*MFN2* and *DRP1*) expression in MSCs (n = 3). Data are expressed as mean ± SD; **p < 0.01, and ns represent p＞0.05.


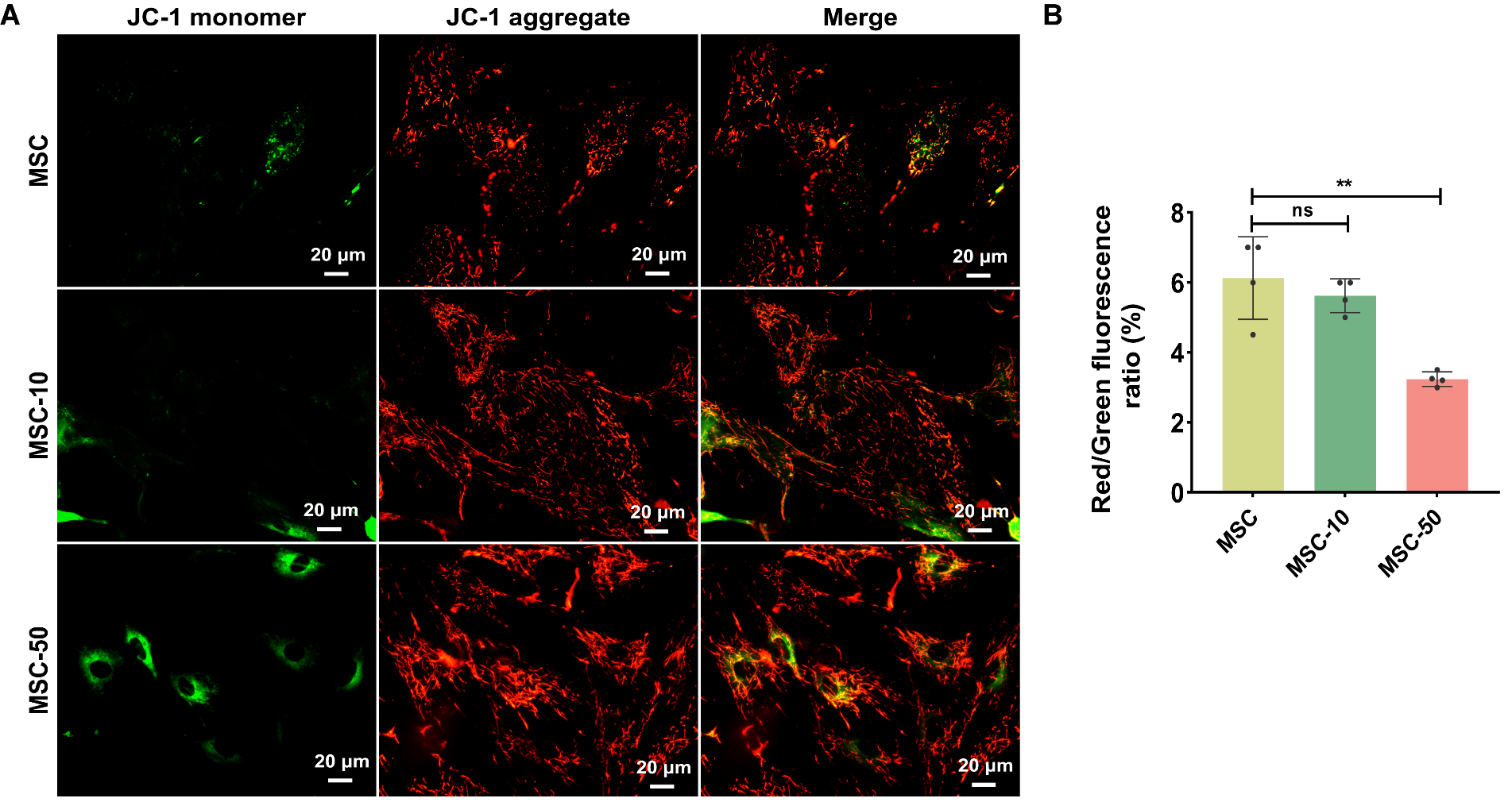


**Figure S3.** Mitochondrial membrane potential was detected by JC-1 fluorescent probe. (A) Representative fluoresce images of JC-1 staining. Scale bar, 20 μm. (B) Mitochondrial membrane potential was calculated by percentage of red to green fluorescent ratios (n = 4 from three biologically independent cultures). Data are expressed as mean ± SD; **p < 0.01, and ns represent p＞0.05.

**
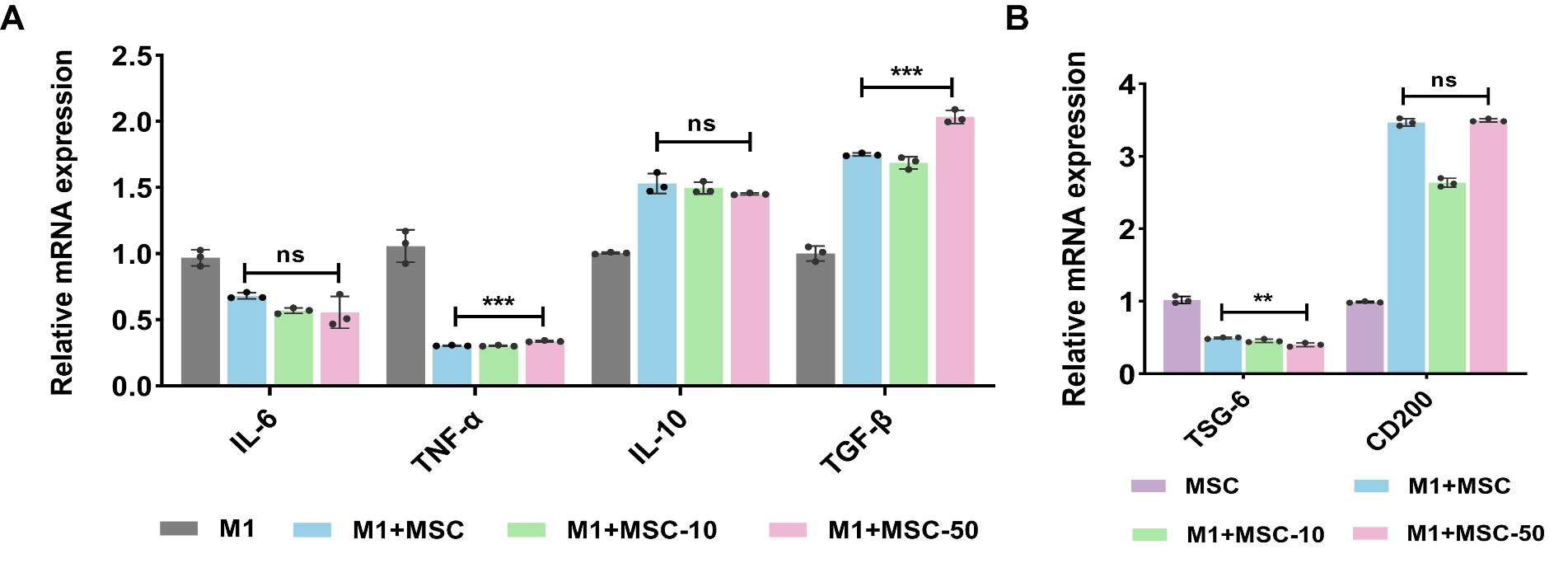
**

**Figure S4.** Cell-to-cell contact coculture system of MSC or N_3_-MSC with M1 macrophages. (A) mRNA expression levels of proinflammatory and anti-inflammatory cytokines in co-culture system (n= 3). (B) mRNA expression of TSG-6 and CD200 in MSCs or N_3_-MSCs co-cultured with M1 macrophages (n = 3). Data are expressed as mean ± SD; **p < 0.01, ***p < 0.001 and ns represent p＞0.05.


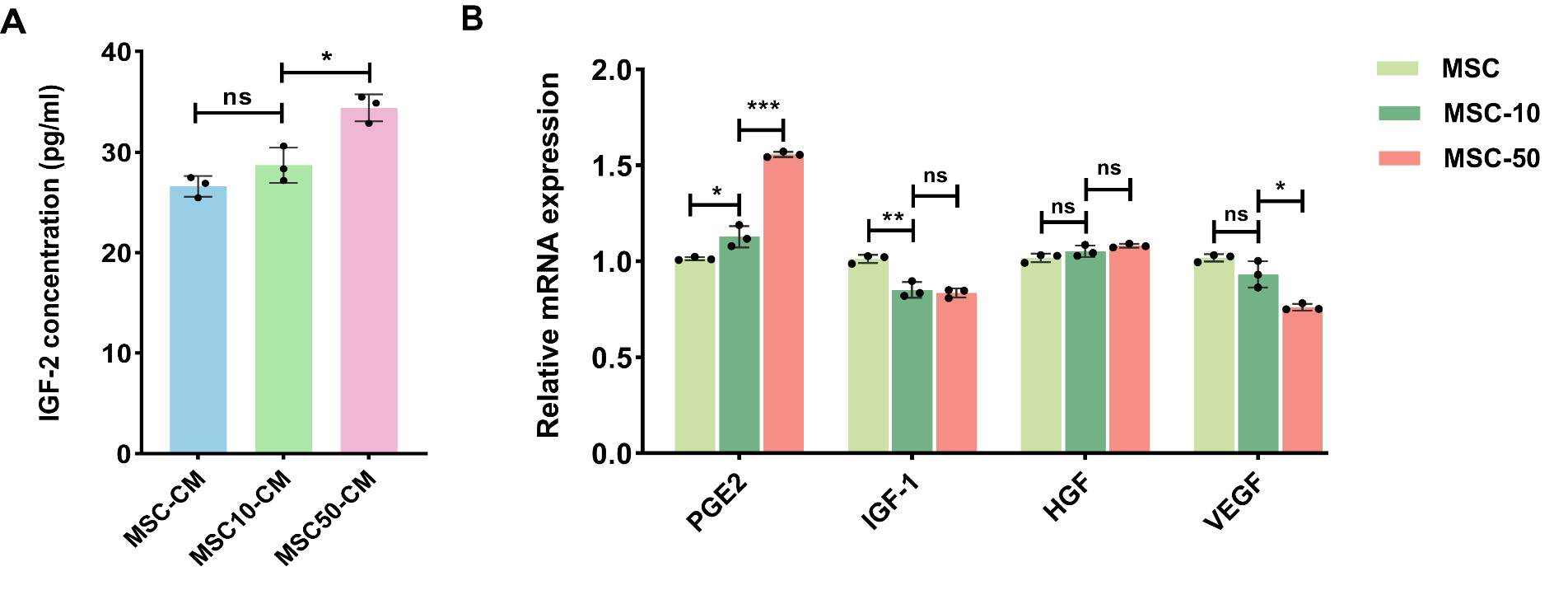


**Figure S5.** Secretion of IGF-2 protein and mRNA levels of bioactive factors in MSC and N_3_-MSC. (A) ELISA was employed to quantify IGF-2 protein levels in MSC-CM and N_3_-MSC-CM (n = 3). (B) mRNA expression levels of bioactive factors in MSCs treated with 10 μM or 50 μM Ac_4_ManNAz (n = 3). Data are expressed as mean ± SD; *p < 0.05, ***p < 0.001, and ns represent p＞0.05.

**
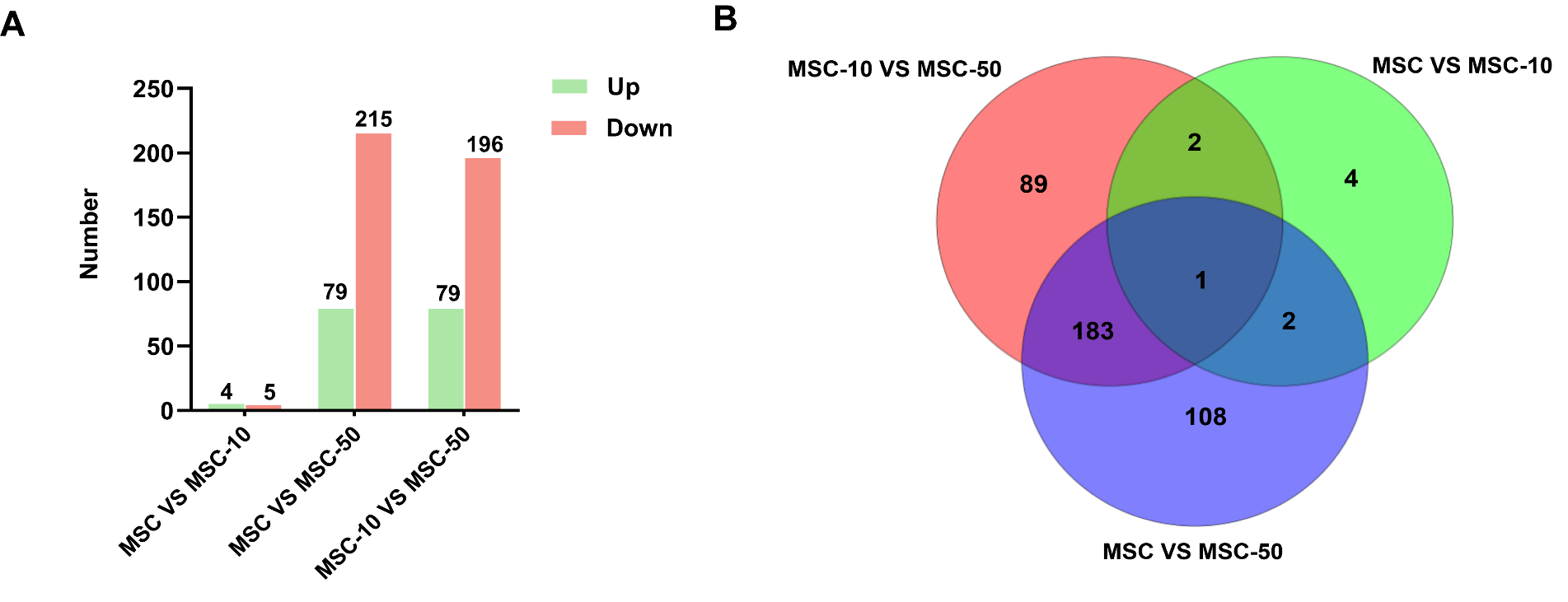
**

**Figure S6.** Visualization and quantification of differentially expressed genes among groups. (A) Number of differentially expressed genes in comparisons between MSC vs. MSC-10 groups, MSC vs. MSC-50 groups and MSC-10 vs. MSC-50 groups. (B) Venn diagrams show differential and common expressed genes among the groups.


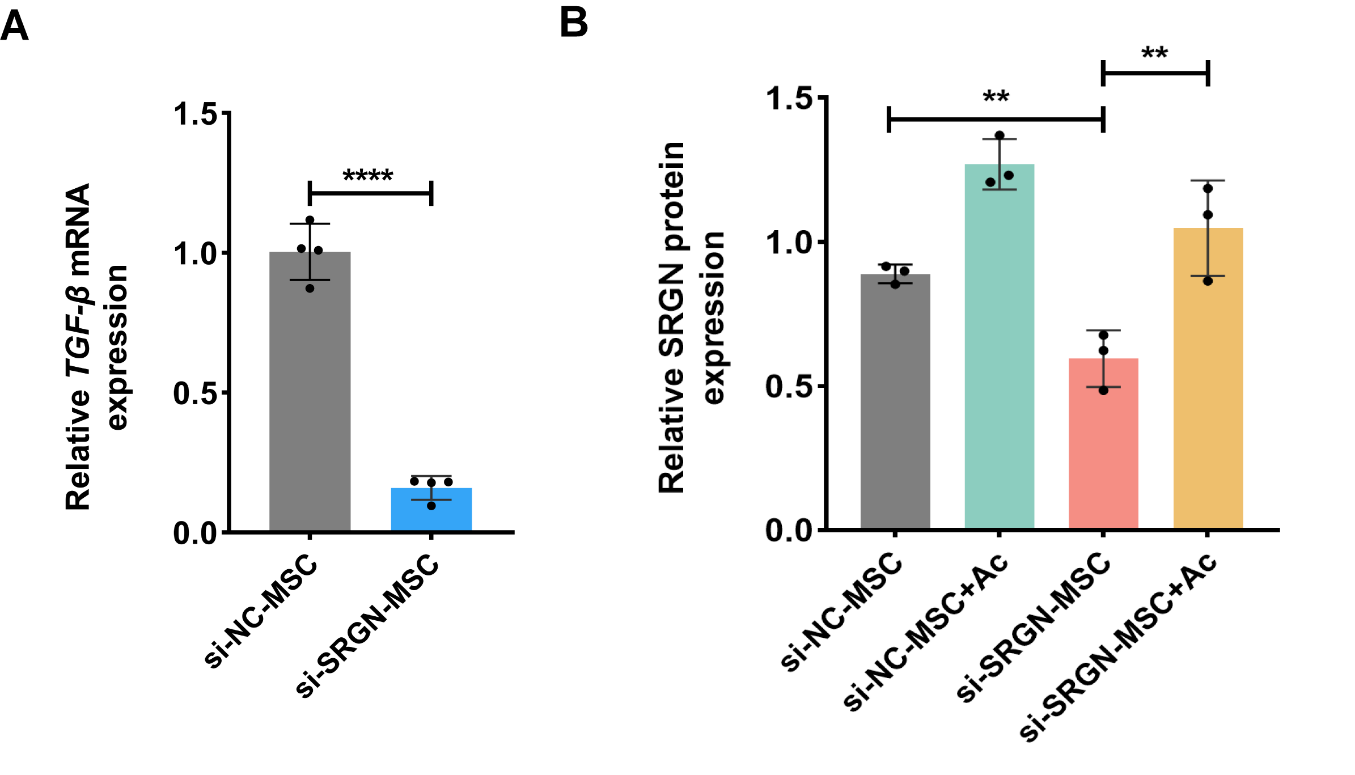


**Figure S7**. TGF-β mRNA expression following si-SRGN treatment and SRGN protein levels in rescue experiments. (A) TGF-β mRNA expression level after si-SRGN treatment (n = 4). (B) Statistical analysis of the gray values of SRGN protein expression in the rescue experiment (n = 3). Data are expressed as mean ± SD; *p < 0.05, **p < 0.01, and ****p ＜ 0.0001.


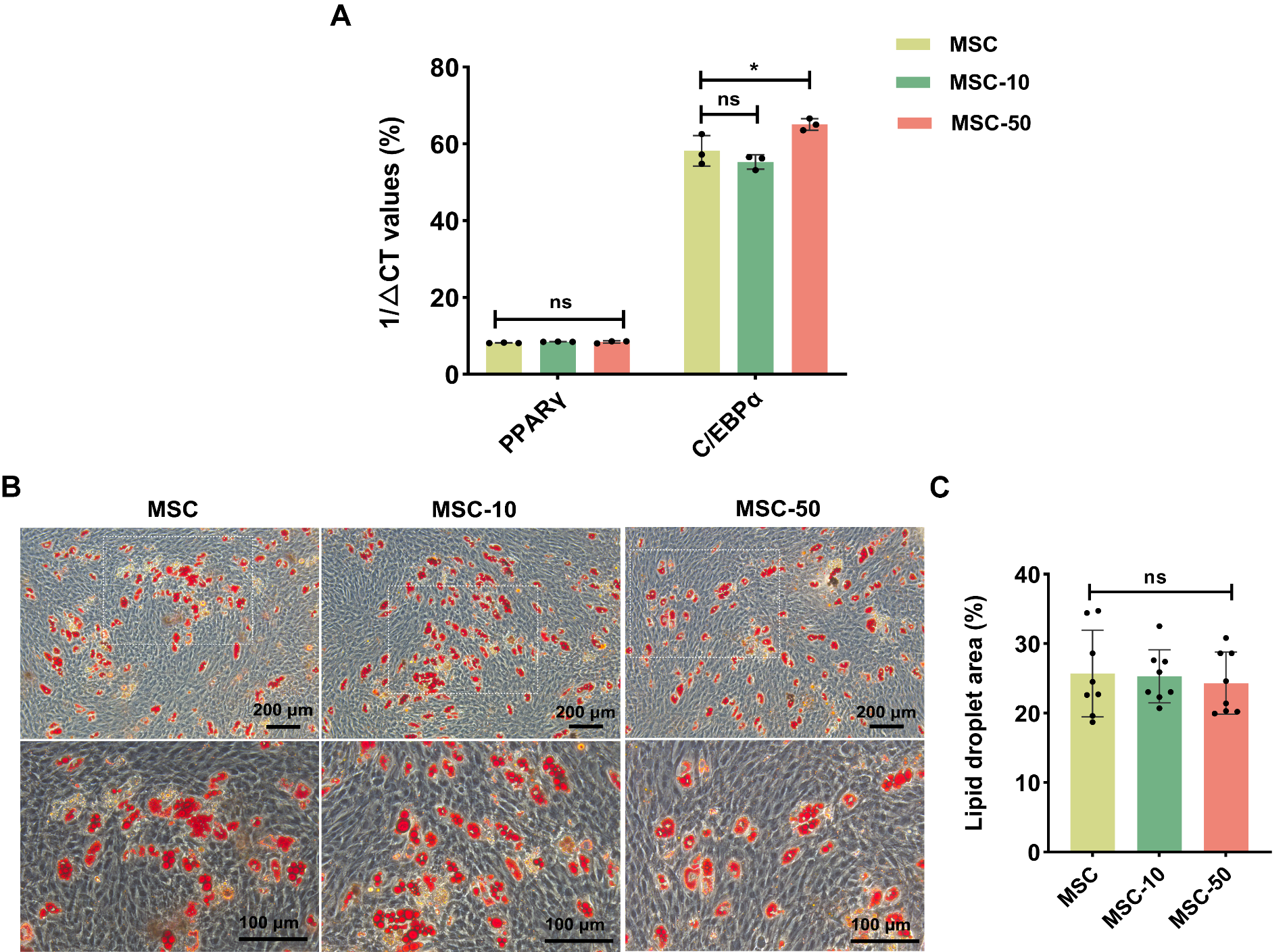


**Figure S8.** The adipogenic differentiation potential of MSCs treated with Ac_4_ManNAz. (A) mRNA expression levels of adipogenic differentiation markers in MSCs treated with 10 μM or 50 μM Ac_4_ManNAz (n = 3). (B) Representative Oil Red O staining images of MSC differentiation. Scale bar, 200, and 100 μm (magnification). (C) Statistical analysis of lipid droplet area in each group (n = 8 from three biologically independent cultures). Data are expressed as mean ± SD; *p < 0.05, and ns represent p﹥0.05.


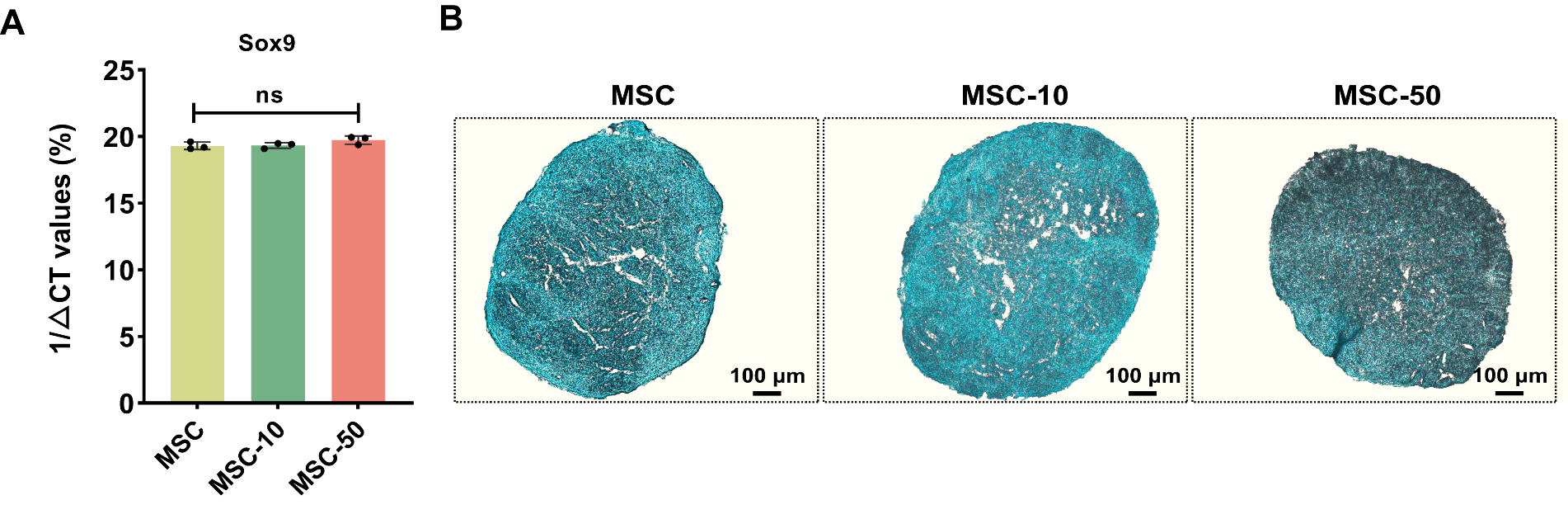


**Figure S9.** The chondrogenic differentiation potential of MSCs treated with Ac_4_ManNAz. (A) mRNA expression levels of chondrogenic differentiation markers in MSCs treated with 10 μM or 50 μM Ac_4_ManNAz (n = 3). (B) Representative Alcian Blue staining images of MSC differentiation. Scale bar, 100 μm. Data are expressed as mean ± SD; ns represent p﹥0.05.


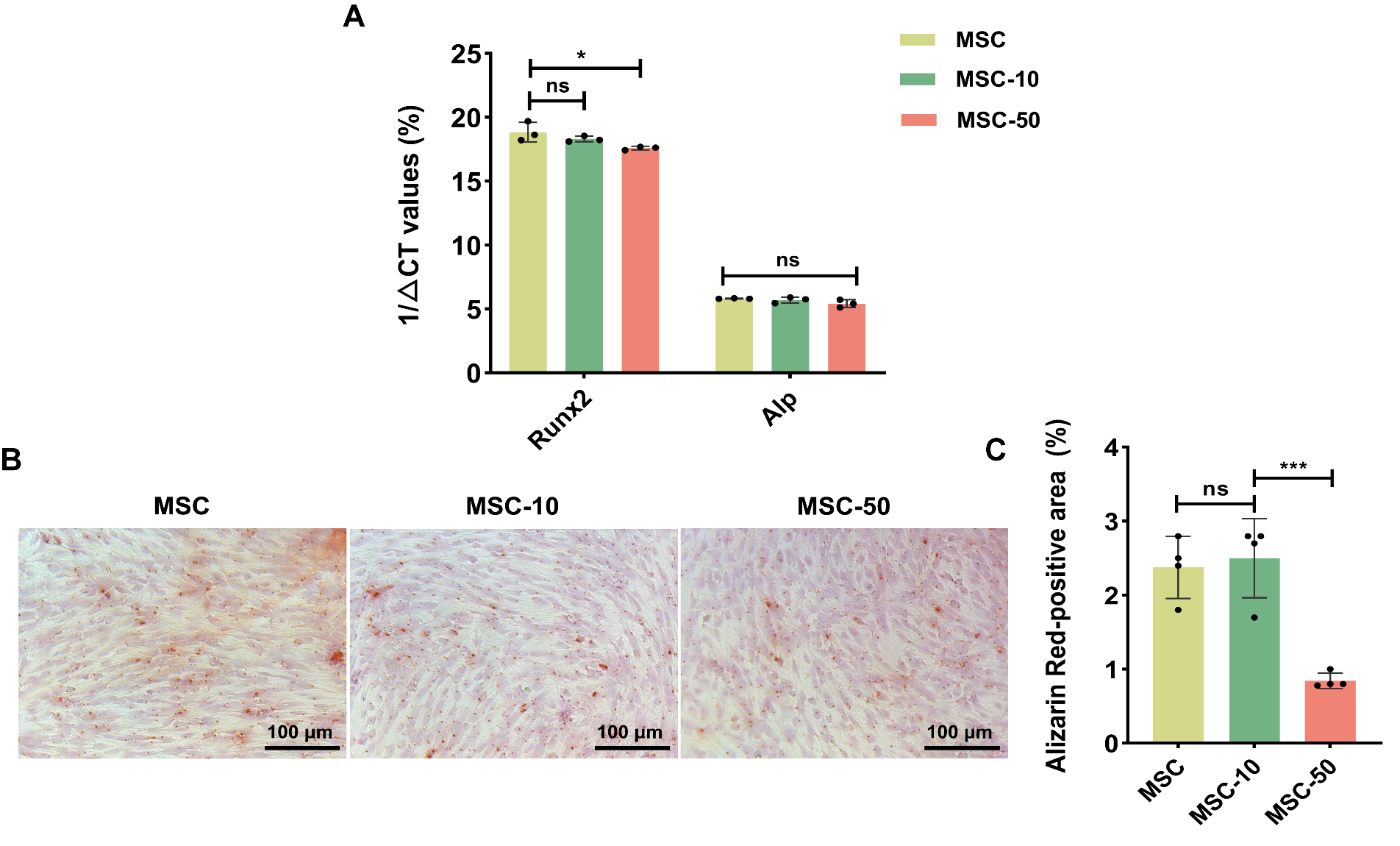


**Figure S10.** The osteogenic differentiation potential of MSCs treated with Ac_4_ManNAz. (A) mRNA expression levels of osteogenic differentiation markers in MSCs treated with 10 μM or 50 μM Ac_4_ManNAz (n = 3). (B) Representative ARS staining images of MSCs differentiation. Scale bar, 100 μm. (C) Statistical analysis of alizarin red-positive area in each group (n = 4 from three biologically independent cultures). Data are expressed as mean ± SD; *p < 0.05, ***p ＜ 0.001, and ns represent p﹥0.05.
